# Efficient synthesis 1,4-cyclohexanedicarboxaldehyde by an engineered alcohol oxidase

**DOI:** 10.1186/s40643-022-00570-y

**Published:** 2022-08-13

**Authors:** Yaqi Cheng, Wei Song, Xiulai Chen, Cong Gao, Jia Liu, Liang Guo, Meng Zhu, Liming Liu, Jing Wu

**Affiliations:** 1 School of Life Sciences and Health Engineering, Jiangnan University, 1800 Lihu Road, Wuxi, 214122 China; 2 State Key Laboratory of Food Science and Technology, Jiangnan University, Wuxi, 214122 Jiangsu China; 3 Wuxi Acryl Technology Co., Ltd, Wuxi, 214122 China

**Keywords:** 1,4-Cyclohexanedicarboxaldehyde, Alcohol oxidase, Primary alcohol oxidation reaction, Protein engineering

## Abstract

In this study, we selected and engineered a flavin adenine dinucleotide (FAD)-dependent alcohol oxidase (AOX) to produce 1,4-cyclohexanedicarboxaldehyde (CHDA), an initial raw material for spiral compounds, from 1,4-cyclohexanedimethanol (CHDM). First, the structure of alcohol oxidase from *Arthrobacter cholorphenolicus* (*Ac*CO) was analyzed, and the mechanism of *Ac*CO-catalyzed primary alcohol oxidation was elucidated, demonstrating that the energy barrier of the hydride (H^−^) transfer (13.4 kcal·mol^−1^ and 20.4 kcal·mol^−1^) decreases the catalytic efficiency of the primary alcohol oxidation reaction. Therefore, we designed a protein engineering strategy to adjust the catalytically active conformation to shorten the distance of hydride (H^−^) transfer and further decreased the core energy barrier. Following this strategy, variant W4 (S101A/H351V/N378S/Q329N) was obtained with 112.5-fold increased catalytic efficiency to produce CHDA compared to that of the wild-type strain. The 3 L scale preparation of CHDA reached a titer up to 29.6 g·L^−1^ with a 42.2% yield by an *Escherichia coli* whole-cell catalyst, which demonstrates the potential of this system for industrial application.

**Graphical Abstract:**

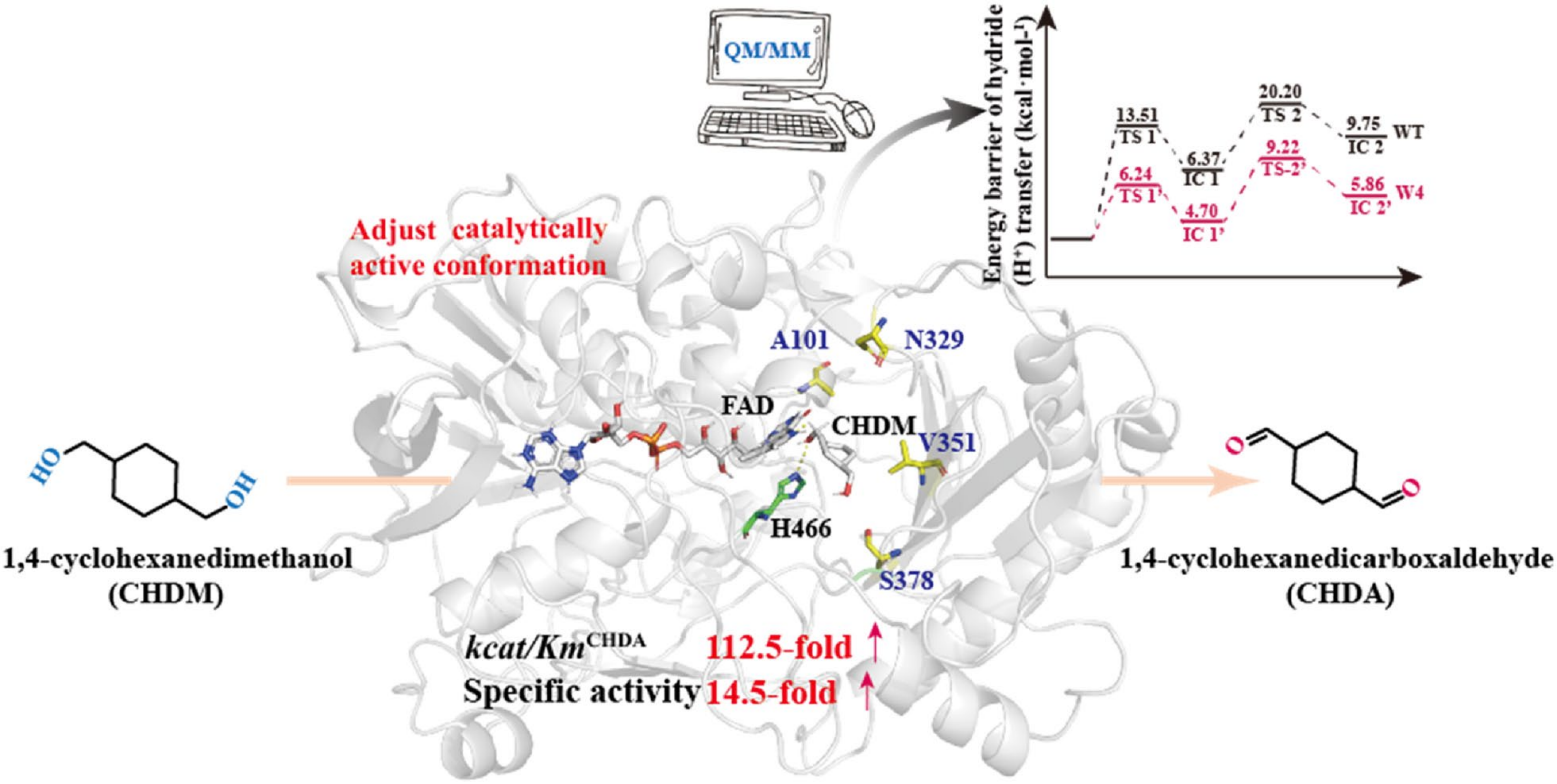

**Supplementary Information:**

The online version contains supplementary material available at 10.1186/s40643-022-00570-y.

## Introduction

1,4-Cyclohexanedicarboxaldehyde (CHDA) is a cyclohexane derivative that contains two aldehyde groups, which is used as the initial raw material in the organic, pharmaceutical, and material fields (Akbulut et al. 2013; Kwit et al. 2007; Pellegrino et al. 2012). For example, CHDA is widely used as a thermotropic liquid crystal module for optical display and screen manufacturing (Feuerbacher et al. 1999). CHDA is commonly synthesized by a hydrogenation reaction (Zhao et al. 2017), hydroformylation reaction (Miao et al. 2021), and primary alcohol oxidation reaction with dimethyl 1,4-cyclohexanedicarboxylate, 3-cyclohexene-1-carboxaldehyde, and 1,4-cyclohexanedimethanol (CHDM) as the substrate, respectively. Among them, the primary alcohol oxidation reaction from CHDM to CHDA is efficient without by-product formation, and is thus a widely used process, which uses a chemical oxidant (Silbert et al. 2019) and transition metal as a catalyst (Asadi et al. 2014; Zhou et al. 2016).

The primary alcohol oxidation reaction is an important reaction in organic synthesis to convert alcohols to aldehydes, including aliphatic aldehydes, benzyl aldehydes, and allyl aldehydes, which are the essential building blocks for fine chemicals, pharmaceuticals, fragrances, and food ingredients (Kunjapur and Prather 2015; Wu et al. 2021; Krishnan et al. 2020). There are three types of catalysts to catalyze primary alcohol oxidation, including typical chemical oxidants, homogeneous catalysts, and heterogeneous catalysts. However, typical chemical oxidants, such as Cr (VI) (Elmaaty and Castle 2005), Mn (VII) (Hossain and Shyu 2010), heptavalent iodine (Lee and Lee 2009), and dimethyl sulfoxide (DMSO)-coupled reagents (Sheldon 2012), exhibit disadvantageous properties of low atomic utilization, low safety, and high cost. Homogeneous catalysts mainly use the Cu/2,2,6,6-tetramethylpiperidinooxy (TEMPO) system for the reaction and can use air-oxidized alcohol to produce the corresponding aldehyde without an additional base; however, the catalyst cannot be recycled, which results in higher production costs (Marais et al. 2021). Heterogeneous catalysts involving metal and non-metal catalysts, such as Ru (Yamaguchi and Mizuno 2002), Pd (Pillai and Sahle-Demessie 2004), Au (Abad et al. 2006), SiO_2_ (Raynal et al. 2014), and C_3_N_4_ (Cruz et al. 2016), have excellent stability and dispersibility; however, the preparation of metallic catalysts is very expensive and the alcohol is more readily peroxidized by non-metallic catalysts. In addition to chemical catalysts, the primary alcohol oxidation reaction can be catalyzed by biocatalysts, such as laccases (E.C.1.10.3.2) (Bassanini et al. 2021), alcohol dehydrogenases (ADHs, E.C.1.1.1) (Zhou et al. 2020), and alcohol oxidases (AOX, E.C.1.1.3) (Tjallinks et al.^2021^; Mattey et al. 2021). This method has great application prospect because of the mild reaction conditions, convenient operations, and high regioselectivity.

In this study, we selected alcohol oxidase from *A. cholorphenolicus* (*Ac*CO) as the biocatalyst to convert CHDM to CHDA and elucidated the catalysis mechanism. Based on this catalysis mechanism, we established a directed evolution strategy to adjust the catalytically active conformation to further decrease the energy barrier of hydride (H^−^) transfer and obtained an optimal variant, W4 (S101A/H351V/N378S/Q329N), with 112.5-fold higher catalytic activity to produce CHDA. The CHDA titer reached 29.6 g·L^−1^ with a 42.2% yield in a 3 L FAD-dependent *Ac*CO-catalyzed process.

## Materials and methods

### Materials

The expression plasmid pET28a(+) and the host strain *Escherichia coli* BL21(DE3) were purchased from Novagen (Madison, WI, USA). The restriction enzymes (*Sac*I, *Sal*I, *BamH*I, and *EcoR*I) and PrimerSTAR polymerase were purchased from TaKaRa Biomedical Technology (Dalian, China). KOD polymerase and the one-step cloning kit were purchased by Talen Biomedical Technology (Shanghai, China) and Vazyme (Nanjing, China), respectively. *Ac*CO, *Ss*ADH, *Brs*ADH, GOase, and HlADH were synthesized by Talen (Shanghai, China) with codon optimization, and the enzymes from four different sources (*Rs*AO, *Tv*L, *Bs*L, and *Tvi*L) were cloned from the corresponding strains (Additional file 1: Table S1). All other chemicals and solvents were obtained commercially.

### Construction of recombinant strains

Each of the *Ac*CO, *Ss*ADH, *Brs*ADH, GOase, HlADH, *Rs*AO, *Tv*L, *Bs*L, and *Tvi*L genes was inserted into the pET28a(+) using corresponding restriction sites (*BamH*I*, EcoR*I, *Sac*I*,* or *Sal*I) to construct the recombinant strains, respectively. All primers used in this study are listed in Additional file 1: Table S2. Next, the target gene fragment was recombined into the linearized vector using the one-step cloning kit to obtain recombinant strains. Finally, the resultant PCR products were transformed into *E. coli* BL21(DE3) cells for DNA sequencing (GENEWIZ, China) or enzyme purification.

### Mutant library construction and screening

The primers used for constructing the variants of *Ac*CO are summarized in Additional file 1: Table S3*. Ac*CO (GenBank Assembly: ACL41642.1) from *A. cholorphenolicus* was inserted into pET-28a(+) using the restriction sites *BamH*I and *EcoR*I for expression in *E. coli* BL21(DE3). NNK site-saturation mutagenesis and iterative saturation mutation of variants were carried by whole-plasmid polymerase chain reaction (PCR) protocol (KOD polymerase), and the PCR product was digested by *Dpn*I and then transformed into *E. coli* BL21(DE3) cells for further screening. To high-efficiently screen the positive variants from 96-well plates, the reaction mixture was prepared and extracted by dichloromethane. Next, the underlying organic phase was separated, and the titer was detected by GC. The variants which performed higher yield of CHDA than wild-type (WT) enzyme were selected to secondary screening in flasks. The detailed steps are described in the Additional file 1: additional methods.

### Homology modeling and MD simulations

For the MD simulation: the 3D structures of *Ac*CO and its mutants were constructed based on the reported X-ray crystal structure of *Ag*CO from *Arthrobacter globiformis* (PDB ID: 3LJP) by homology modeling (https://swissmodel.expasy.org/), and the protonation states were assigned based on the Protein Preparation module of the Schrodinger software suit. The CHDM and 4-(hydroxymethyl)cyclohexanecarboxaldehyde (HMCA) molecules were docked to the reference ligand position around the FAD molecule with the Glide standard precision protocol. The docked structures were prepared through the CHARMM–GUI input generator for atomistic molecular dynamics (MD) simulations performed in the GROMACS (version 2020.6) simulation package (Hess et al. 2008), using the CHARMM 36 force field (Best et al. 2012) and the TIP3P water model. The protein was initially placed in the center of a cubic box with a length of 9.3 nm and each side extending 1.5 nm from the protein surface for the solvation of more than 20,000 water molecules. After thousands of steps of energy minimization, the systems were equilibrated with position restraints on the protein backbone and the heavy atoms of the ligand (CHDM and HMCA) with a restraining constant of 400 kJ·mol^−1^. Further equilibration runs of 10 ns were performed at the target temperature of 300 K and target pressure of 1.0 atm using the Nose–Hoover and Parrinello–Rahman methods, respectively. Finally, the production MD simulations under the NPT ensemble were extended for 100 ns. An integration time step of 2 fs and a cutoff scheme of 1.2 nm were implemented for the non-bonded interactions, and the Particle Mesh Ewald (Essmann et al. 1995) method with a Fourier spacing of 0.1 nm was applied for the long-range electrostatic interactions. All covalent bonds with hydrogen atoms were constrained using the LINCS algorithm (Hess et al. 1997).

### QM/MM calculations

The representative structures were extracted from the MD trajectories for subsequent QM/MM calculations using the QSite simulation package (QSite, version 6.7, Schrödinger, LLC, New York, NY, 2015). The electronic embedding scheme was used with hydrogen capping on the C1’ and C2’ atoms in FAD to define the QM region. The side chain of H466 and the free ligand of CHDM, HMCA, and CHDA were also included in the QM region to account for the proton transfer effects. The functional hybrid B3LYP was used with the basis set of 6–31 + G** for the geometric optimization of the QM region.

### Enzyme purification

*Ac*CO, GOase, *Rs*AO, *Ss*ADH, *Brs*ADH, HlADH, *Tv*L, *Bs*L, and *Tvi*L were individually overexpressed and purified from *E. coli* BL21(DE3) with pET28a(+) plasmid (the detailed steps of fermentation culture are given in the Additional file 1: additional methods). The cells were collected after fermentation culture and resuspended in Tris–HCl buffer (pH 8.0). Then, the cell suspensions were fragmented with a high-pressure homogenizer at 4 °C and centrifuged at 12,000×*g* for 30 min. Finally, the recombinant strains were purified by the ÄKTA pure system (GE Healthcare).

### Enzyme assay

The specific activity of *Ac*CO or its variants was determined by measuring the initial rate of production of CHDA or HMCA by GC under the following conditions: 10 μM purified *Ac*CO or variants, 0.1 g·L^−1^ catalase, 5% (v/v) DMSO, and 5 mM HMCA or CHDM in 1 mL air-saturated potassium phosphate buffer (100 mM, pH 8.0) at 30 °C for 30 min. The reaction mixture was extracted by dichloromethane, centrifuged at 12,000×*g* for 10 min, and the samples were analyzed by GC. One unit of *Ac*CO or variants activity (U) was calculated as the amount of enzyme producing 1 μM of CHDA or HMCA in 1 min. All experiments were repeated three times. The enzyme assay of the other sources of enzymes are described in the Additional file 1: additional methods.

### Determination of kinetic parameters

The kinetic parameters of purified *Ac*CO or its variants toward CHDA or HMCA were performed at 30 °C in potassium phosphate buffer (100 mM, pH 8.0), which contained 0.1 g·L^−1^ catalase, 5% (v/v) DMSO, with CHDM or HMCA concentration varied from 1 to 100 mM. When the conversion was less than 10%, the reaction mixture was extracted by dichloromethane, centrifuged at 12,000×*g* for 10 min and the production of CHDA or HMCA was determined by GC. All experiments were repeated three times. The *K*_M_ and *k*_cat_ values were calculated by nonlinear regression according to the Michaelis–Menten equation using Origin software.

### Biocatalytic reaction on analytical scale

A 1 mL scale biocatalytic reaction mixture containing whole cell catalyst (30 g·L^−1^ wet *E. coli*), CHDM (5 mM), DMSO (5% v/v), catalase (0.1 g·L^−1^), and air-saturated potassium phosphate buffer (100 mM, pH 8.0) was shaken at 30 °C for 12 h. Then, the reaction mixture was extracted by dichloromethane and centrifuged at 12,000 × *g* for 10 min. The titer of CHDA and HMCA was determined by GC. All experiments were repeated three times. The detailed steps of fermentation culture can be seen in the Additional file 1: additional methods).

### Production of CHDA on a 3 L scale

The conversion experiments were carried out in a 3 L bioreactor with 1 L working volume. For 3 L scale production of CHDA, the whole cell catalyst (30 g·L^−1^ wet *E. coli*) was evenly distributed in a 1 L air-saturated potassium phosphate buffer (100 mM, pH 8.0) containing CHDM (0.5 M, 72 g·L^−1^), DMSO (5% v/v), and catalase (0.1 g·L^−1^) was added to a 3 L fermenter, where the reaction mixture was stirred at 30 °C. Take reaction mixture (500 μL) and determine the titer of CHDA and HMCA by GC at the end of the reaction. The detailed steps of fermentation culture can be seen in the Additional file 1: additional methods.

### Analytical methods

The titer of HMCA and CHDA were measured by SHIMADZU GC-2030 (GC) with a DB-5 column (30 m × 0.25 mm × 0.25 μm; Daicel Corporation, Japan) and nitrogen as the carrier gas. The carrier gas flow rate was 1.0 mL·min^−1^ and no shunt, injector temperature was 250℃; the initial column temperature was 80 °C for 2 min, and the temperature was increased to 250 °C at 10 °C min^−1^. The samples were filtered through a 0.22 μm organic membrane and then analyzed by GC.

## Results

### Role of *Ac*CO in the transformation from CHDM to CHDA

**Scheme 1:**
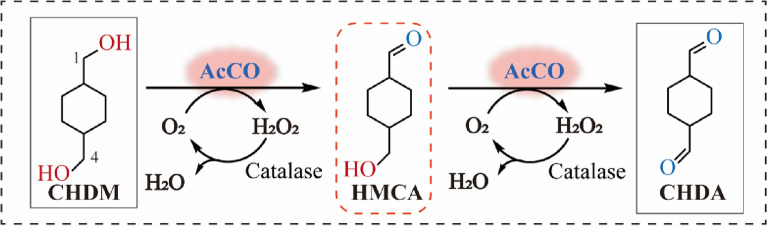
Aim of this study: schematic representation of CHDA biosynthesis from CHDM

The synthesis of CHDA from its structural analog CHDM through a primary alcohol oxidation reaction can be catalyzed by AOX, ADH, and laccases. However, the regioselectivity of CHDM poses a major challenge for executing this reaction, as these enzymes could regioselectively oxidize one hydroxyl group of CHDM to produce the intermediate (HMCA), thereby changing the chemical properties and hindering further oxidation of another hydroxyl group (Additional file 1: Fig. S1). To address this challenge, we selected three different strains for each enzyme to evaluate the specific activity of those enzymes toward CHDA. Among them, the FAD-dependent AOX from *A. cholorphenolicus* (*Ac*CO) and ADH from *Sulfolobus solfataricus* (*Ss*ADH) displayed the highest ability to produce CHDA (specific activity of 0.11 ± 0.026 and 0.094 ± 0.015 U·g^−1^, respectively) (Additional file 1: Tables S4–S6). However, *Ss*ADH requires an NAD^+^/cofactor-recycling system, and the equilibrium is unfavorable for the preparation of the aldehyde; therefore, *Ac*CO was selected for further study, given that it uses oxygen (air) for cofactor regeneration and the reaction is irreversible (Scheme 1). Furthermore, *Ac*CO can be heterologously expressed in *E. coli* [many other AOXs (Cleveland et al. 2021; Mathieu et al. 2020) and ADHs (Zhao et al. 2009) require eukaryotic expression systems] and the recombinant enzyme exhibited higher yield for the production of CHDA, with a ratio of CHDA to HMCA (R_CHDA_/_HMCA_) of 0.25 (Additional file 1: Figs. S2 and S3).

**Fig. 1:**
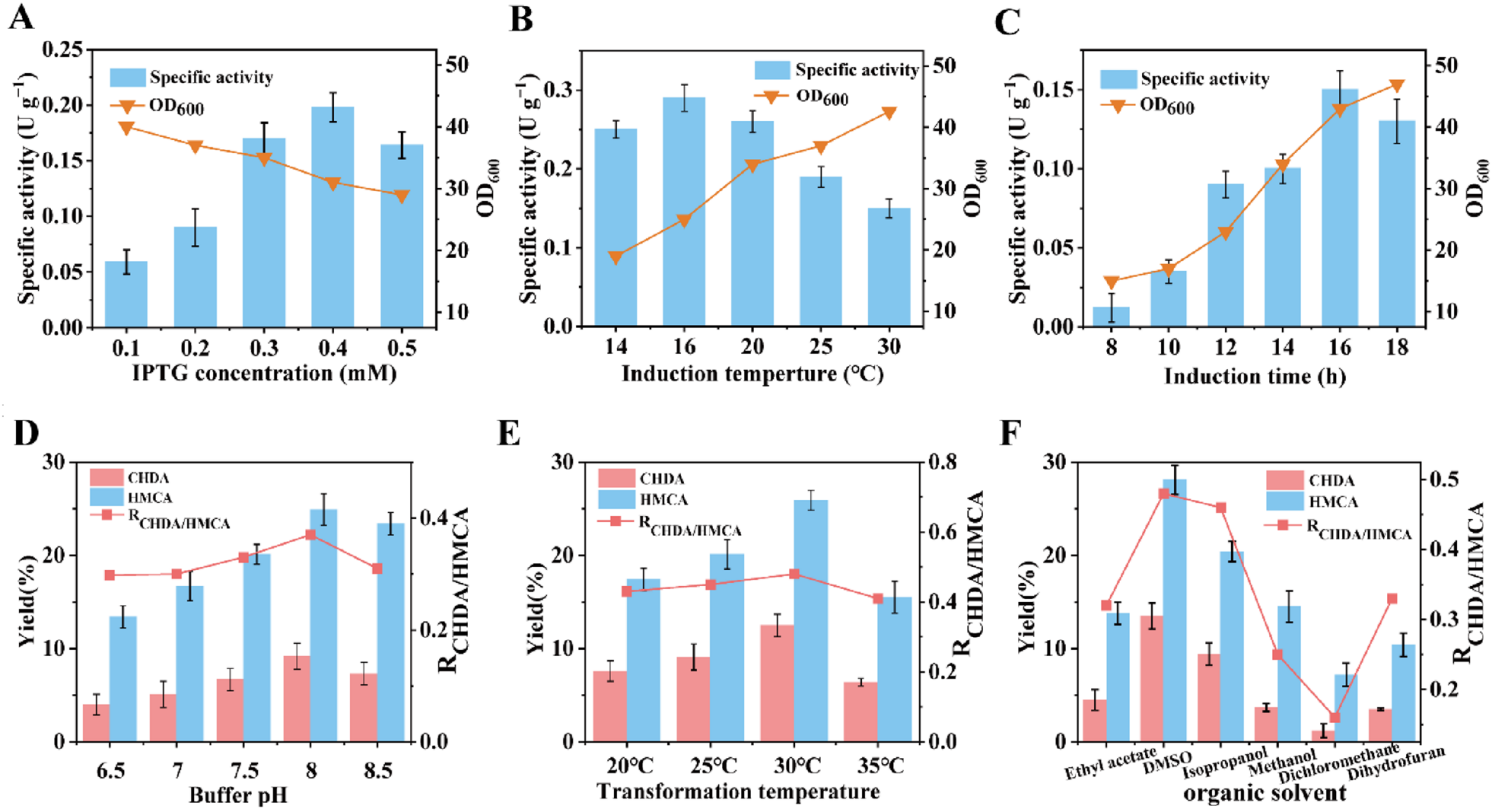
Optimizations of the wild type *Ac*CO in induction and conversion process. **A** Effect of induction time on the specific activity and cell growth of the WT. **B** Effect of IPTG concentrations on the specific activity and cell growth of the WT. **C** Effect of induction temperature on the specific activity and cell growth of the WT. **D** Effect of buffer pH on the R_CHDA/HMCA_ and the yield of CHDA and HMCA. **E** Effect of transfer temperature on the R_CHDA/HMCA_ and the yield of CHDA and HMCA. **F** Effect of organic solvent type on the R_CHDA/HMCA_ and the yield of CHDA and HMCA. The specific activity and yield were obtained through measuring the titer of CHDA and HMCA by GC analysis

To increase the primary alcohol oxidation activity of *Ac*CO, the expression conditions, including the induction temperature, inducer concentration, and induction time, were optimized. As shown in Figs. 1A–C, the specific activity of *Ac*CO toward CHDA increased from 0.11 ± 0.026 U·g^−1^ to 0.29 ± 0.097 U·g^−1^ under the optimum conditions (16 °C for 2.5 h, 0.4 mM IPTG, and induction time of 16 h). Furthermore, the effect of organic solvents, buffer pH (6.5–8.5), temperature (20–35 °C), and organic solvent concentration [0–10% (v/v)] on CHDA production were investigated, and the results are shown in Figs. 1D–F and Additional file 1: Fig. S4. The yield of CHDA increased to 13.5% under the optimum conditions [30 g L^−1^ wet *E. coli*, pH 8.0, 100 mM air-saturated potassium phosphate buffer, 5% (v/v) DMSO, 0.1 g L^−1^ catalase, 30 °C for 12 h]. The yield of CHDA from CHDM slightly increased from 5.0 to 13.5%, although the titer and yield of HMCA from CHDM continued to increase from 1.0 mM to 1.4 mM and from 20.4 to 28.1%, respectively, resulting in an increase of the R_CHDA/HMCA_ to 0.48. Therefore, we determined that further engineering of *Ac*CO was required to increase the selectivity of CHDA and reduce the accumulation of HMCA. Toward this end, we explored the structure of *Ac*CO and the catalytic mechanism of primary alcohol oxidation reaction.

### The catalytic mechanism of *Ac*CO

**Fig. 2:**
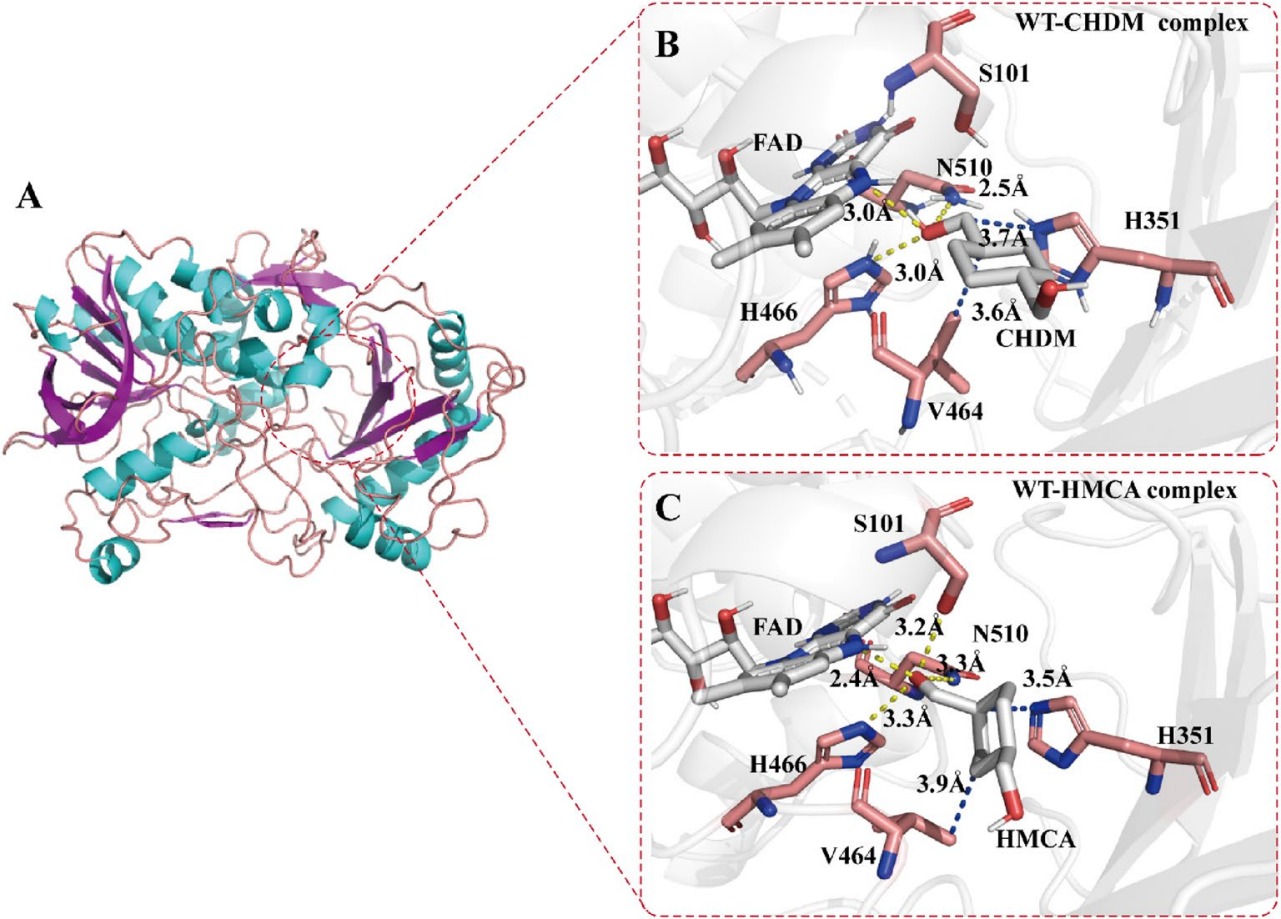
Docking analysis of *Ac*CO (WT). **A** Homology model of *Ac*CO. **B** Docking model of CHDM in *Ac*CO. **C** Docking model of HMCA in *Ac*CO. CHDM, HMCA and FAD are shown in white, residues S101, N510, H466, V464 and H351 are shown in pink. The hydrogen-bond interactions are shown in yellow dash lines and steric interactions are shown in blue dash line

A homology model of *Ac*CO was reconstructed using SWISS–MODEL (https://swissmodel.expasy.org/) based on the crystal structure of choline oxidase from *A. globiformis* (91% sequence identity) (Fig. 2A and Additional file 1: Fig. S5). According to the previous reports on *Ag*CO, H466 plays an important role in the reaction as a catalytic base and FAD is involved in hydride (H^−^) transfer (Smitherman et al. 2015). Docking analysis using the *Ac*CO structural model and CHDM (Fig. 2B) revealed that the contact between CHDM and FAD occurs through an O atom of the 1-Cα-OH, which is at a distance of 3.0 Å from the N(5) atom of FAD. Moreover, in the active center of *Ac*CO, two hydrogen-bond interactions were identified between 1-Cα-OH of CHDM with the side-chain amide of N510 (2.5 Å) and the N^ε2^ atom of H466 (3.0 Å), as well as steric interactions between the C atom of CHDM with the side chains of V464 (3.6 Å) and H351 (3.7 Å), anchoring CHDM in the binding pocket. In this conformation, the cyclopentane portion of CHDM was almost parallel to the FAD isoalloxazine ring, which results in 1-Cα-OH of CHDM being retained on the inside of the binding pocket and close to the active center and 4-Cα-OH of CHDM extending toward to the outside of the binding pocket stay away from the catalytic center; the distance between the 4-Cα-OH with the N(5) atom of FAD and the N^ε2^ atom of H466 is 7.9 Å and 9.8 Å, respectively (Additional file 1: Fig. S6A). These findings indicated that the two Cα-OH groups of CHDM incompletely fit in the binding pocket, and the conformation of CHDM is not beneficial to produce HMCA. Given the accumulation of HMCA, we next analyzed the *Ac*CO and HMCA docking model (Fig. 2C). Four hydrogen-bond interactions were identified between the 1-CHO with the N(5) atom of FAD (2.4 Å), the side-chain amide of N510 (3.3 Å), the N^ε2^ atom of H466 (3.3 Å), and the side-chain hydroxy of S101 (3.2 Å), as well as steric interactions between HMCA with the side chains of V464 (3.9 Å) and H351 (3.5 Å). In this conformation, although the cyclopentane plane of the HMCA generated an angle deflection [the angle (θ) between the 1-CHO of HMCA, the N(5) atom, and the N(10) atom of FAD (θ_N(10)-N(5)-(1-CHO)_) shifted to 95° (the θ_N(10)-N(5)-(1-Cα-OH)_ is 124.1° in the WT-CHDM complex)], the 4-Cα-OH of HMCA was maintained toward the outside of the binding pocket (the distance between 4-Cα-OH of HMCA and the N^ε2^ atom of H466 was 10.0 Å) and the 1-CHO of HMCA was stably buried in the active center (Additional file 1: Fig. S6A, B). We deduced that it is difficult to further oxidize the 4-Cα-OH of HMCA due to the long catalytic distance from the active center, which causes the accumulation of HMCA. To verify this conjecture, MD simulation and QM/MM calculations were performed, the representative snapshot and the change of energy barrier were used to understand the molecular basis of the primary alcohol oxidation reaction.

**Fig. 3:**
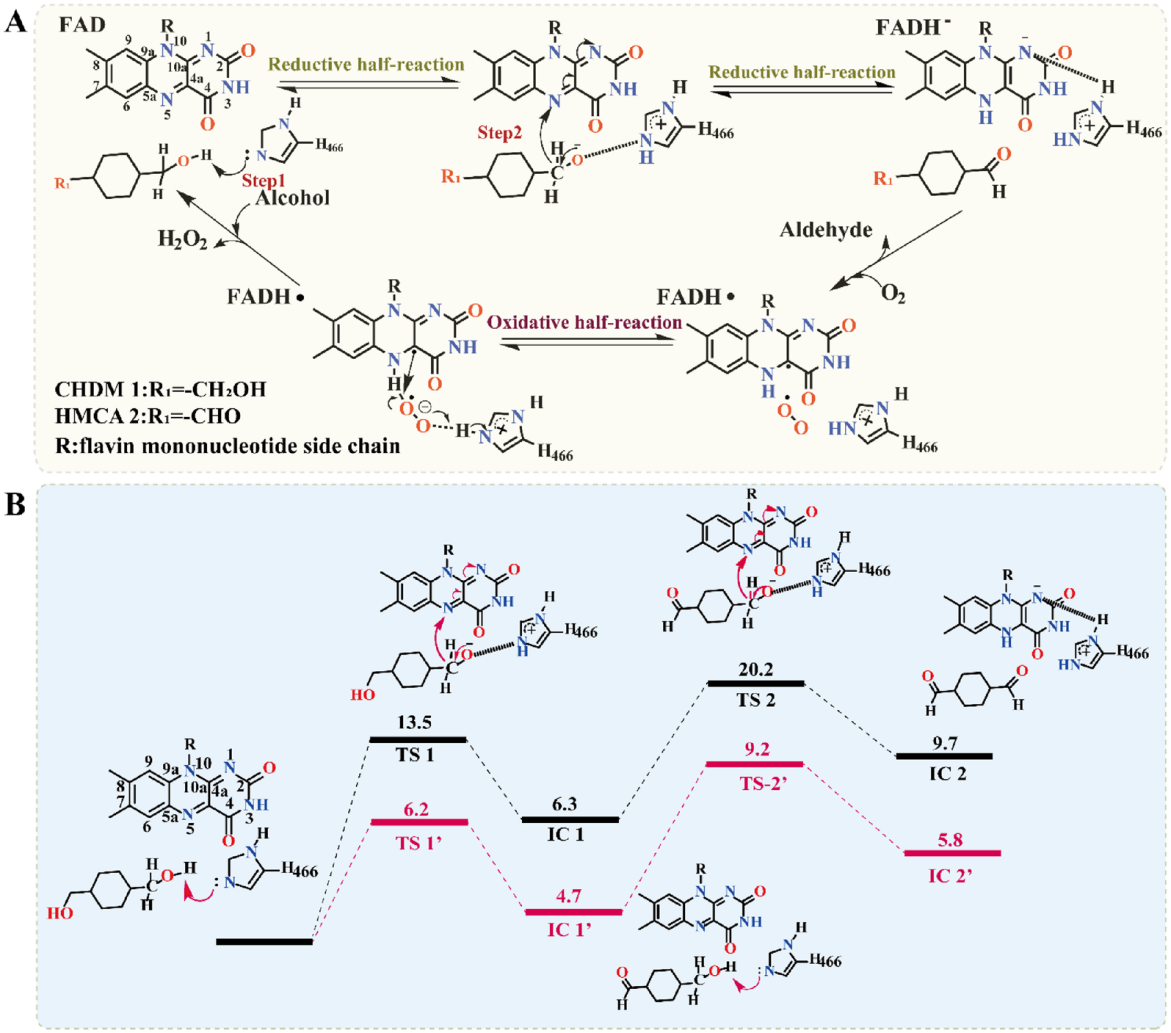
QM/MM calculation of *Ac*CO. **A** Proposed catalytic reactions of *Ac*CO. The proton and hydride (H^−^) transfer from CHDM and HMCA to H466 (N^ε2^) and FAD N(5) is marked as arrows. **B** Relative energy profile (in kcal·mol^−1^) of hydride (H^−^) transfer from CHDM and HMCA, along with schematic drawings of key intermediates involved in the reaction. The WT is shown in black line, variant W4 is shown in red line

**Fig. 4:**
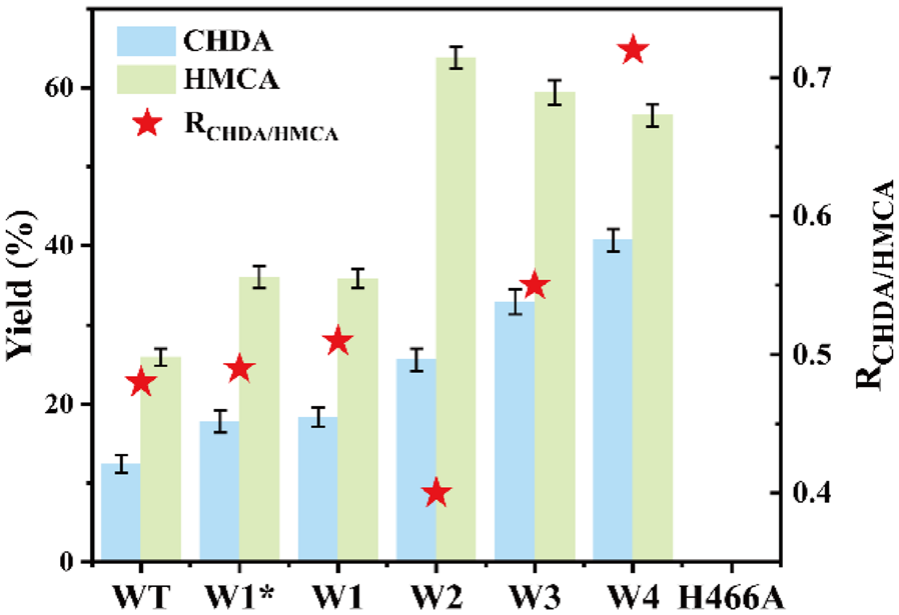
Directed evolution of the *Ac*CO for primary alcohol oxidation of CHDM. Reactions were performed in recombinant *E. coli* (30 g·L^−1^ whole cell catalyst) and 5 mM CHDM in 1 mL air-saturated potassium phosphate buffer (100 mM, pH 8.0) at 30 °C for 12 h (600 rpm). The yield was determined by GC

As shown in Fig. 3A, based on the catalytic mechanism proposed by Gadda’s group (Smitherman et al. 2015; Gadda 2020), His466 should be deprotonated under basic conditions. The QM/MM optimized reactant complex shows that the deprotonated His466 forms a stable hydrogen bond with the Cα-OH of CHDM or HMCA. This result suggested that deprotonated His466 can serve as a basic catalyst during the transfer of the hydride (H^−^) from CHDM or HMCA to FAD. To verify the function of this residue, we mutated H466 to alanine, finding that H466A completely abolished the primary alcohol oxidation activity **(**Fig. 4). Thus, we inferred that *Ac*CO catalyzed CHDM to CHDA which could be divided into two reaction steps: (I) reductive half-reaction, and (II) oxidative half-reaction. In the reductive half-reaction, the deprotonated histidine (H466) side chain attacks the 1-Cα-OH of CHDM and a proton from 1-Cα-OH of CHDM is transferred to the deprotonated histidine side chain (H466). Subsequently, the hydride (H^−^) in 1-Cα-H of CHDM is transferred to the N(5) of FAD isoalloxazine ring to form FADH^−^, this step involves a 13.5 kcal·mol^−1^ energy barrier (Fig. 3B). Finally, 1-Cα-OH of CHDM is oxidized to aldehyde, accompanied by HMCA synthesis. In the oxidative half-reaction, the hydride (H^−^) of C4α-peroxy in FADH^−^ and a proton in H466 are transferred to the O-atom of oxygen, to regenerate FAD and release H_2_O_2._ Subsequently, 4-Cα-OH of HMCA is again attacked by H466, and the hydride (H^−^) is transferred from 4-Cα-H of HMCA to the N(5) of FAD isoalloxazine ring; this step involves a 20.2 kcal·mol^−1^ energy barrier and is accompanied by the synthesis of CHDA (Fig. 3B). In this catalytic mechanism, the second but most important step is the transfer of hydride (H^−^) from the CHDM and HMCA to the N(5) of FAD isoalloxazine ring. During this step, 1-Cα-H of CHDM executes the hydride (H^−^) transfer and to form HMCA, and 1-Cα-H of HMCA again transfers the hydride (H^−^) to FAD isoalloxazine ring to ultimately synthesize the final product CHDA. Therefore, the energy barrier of the second step is directly related to product formation, which determines the efficiency of the primary alcohol oxidation reaction.

However, the QM/MM optimized reactant complex showed that the hydride (H^−^) transfer of CHDM and HMCA leads to an energy barrier of 13.5 kcal·mol^−1^ and 20.2 kcal·mol^−1^ in the second step, respectively. Meanwhile, the energy barrier of HMCA is much higher than that of CHDM in the hydride (H^−^) transfer process, which makes it more difficult to oxidize 4-Cα-OH of HMCA to form CHDA. Therefore, we speculated that the energy barrier of this step is the key factor to achieve the low catalytic efficiency of CHDM and the accumulation of HMCA; and how to decrease the energy barrier of hydride (H^−^) transfer is the main issue. According to the representative snapshot of the MD simulation, however, the CHDM and HMCA anchored in the binding pocket represent an unfavorable catalytically active conformation, which is similar to the docking model. In this conformation, the distance between 1-Cα-H of CHDM and the N(5) atom of FAD (D_(1-Cα-H)-N(5)FAD_ = 4.3 Å) and the distance between 4-Cα-H of HMCA and the N(5) atom of FAD (D_(4-Cα-H)-N(5)FAD_ = 9.6 Å) were not suitable for hydride (H^−^) transfer, which may have limited the transfer of hydride (H^−^) and formed a high energy barrier (Fig. 6B, D). This result also confirmed our previous conjecture. Therefore, changing the catalytically active conformation to decrease the distance of hydride (H^−^) transfer was considered to be a potentially effective strategy to reduce the energy barrier.

### Directed evolution of *Ac*CO

Analysis of the interaction of the WT-CHDM and WT-HMCA complex showed that the N(5) atom of FAD, the side-chain amide of N510, and the N^ε2^ atom of H466 have hydrogen-bond interactions with 1-Cα-OH of CHDM and 1-CHO of HMCA, along with an extra hydrogen-bond interaction between 1-CHO of HMCA and S101. The residues H351 and V464 are close to the cyclopentane plane of CHDM and HMCA, which may produce steric interactions with the C atom of CHDM and HMCA (Additional file 1: Fig. S7A, B). Thus, it was speculated that these interactions would firmly fix 1-Cα-OH of CHDM and 1-CHO of HMCA to the active center and produce the steric hindrance to hinder the adjustment of the catalytically active conformation. Therefore, six residues (S101, H351, Y465, N510, I103, and V464) in a radius of 5 Å around 1-Cα-OH of CHDM and 1-CHO of HMCA were selected **(**Additional file 1: Fig. S8A, B) for engineering by NNK site-saturation mutagenesis to change the interactions between the enzyme and CHDM or HMCA. Among them, the activity of two variants, S101A and H351V, increased by 3.3-fold and 4.3-fold to produce CHDA, respectively. Subsequently, variant W2 (S101A/H351V), representing the combination of these two single variants, was constructed, which showed the activity toward CHDA was 2.43 ± 0.13 U·g^−1^, that was 8.3-fold higher than that of WT. As a result, the yield of CHDA increased to 25.6%, and the yield and titer of HMCA increased to 63.8% and 3.19 mM, respectively, leading to an overall decrease of the R_CHDA/HMCA_ to 0.41 and thus a large accumulation of HMCA (Fig. 4, Table 1). The preliminary docking model of the variant W2-HMCA, it demonstrated that the hydrogen-bond interaction between 1-CHO of HMCA and A101 was broken by mutating S101 to A101 (the distance between residue 101 and HMCA increased to 5.1 Å), and when the H351 was mutated to V351, the distance between the side chains of V351 and the C atom of CHDM and HMCA increased to 5.1 Å and 4.9 Å. This shift altered the D_(1-Cα-H)-N(5)FAD_ of CHDM to 2.4 Å, while the distance of nucleophilic attack (D_(1-Cα-OH)-N_^ε2^_H466_) was reduced to 3.0 Å, and the D_(4-Cα-OH)-N_^ε2^_H466_ and D_(4-Cα-H)-N(5)FAD_ of HMCA shifted to 9.0 Å and 7.8 Å, respectively (Additional file 1: Fig. S9). Therefore, we speculated that the conformation was slightly adjusted with the change of interactions, but 4-Cα-OH of HMCA was still not attached to the correct location, so that it was not well fixed to the inside of the pocket, consequently hindering the further oxidation of 4-Cα-OH.

**Table 1:** Kinetic constants of *Ac*CO and its variants in producting CHDA

Strains	^a^Specific activity^CHDA^ (U·g^−1^)	*K*_M_^CHDA^ (mM)	*k*_cat_^CHDA^ (min^−1^)	^b^*k*_cat_/*K*_M_^CHDA^ (mM^−1^·min^−1^)
WT	0.29 ± 0.097	26.81 ± 0.19	0.043 ± 0.013	1.60 × 10^–3^
W1(S101A)	0.95 ± 0.25	20.42 ± 0.12	0.14 ± 0.022	6.86 × 10^–3^
W1*(H351V)	1.24 ± 0.14	16.24 ± 0.11	0.24 ± 0.022	1.48 × 10^–2^
W2(S101A/H351V)	2.43 ± 0.13	14.44 ± 0.46	0.41 ± 0.014	2.84 × 10^–2^
W3(S101A/H351V/N378S)	2.87 ± 0.43	6.81 ± 0.13	0.53 ± 0.12	7.78 × 10^–2^
W4(S101A/H351V/N378S/Q329N)	4.21 ± 0.38	5.24 ± 0.28	0.94 ± 0.043	0.18

^a^The specific activity of CHDA was determined with 10 μM purified *Ac*CO (WT) or its variants and 5 mM HMCA in 1 mL air-saturated potassium phosphate buffer (100 mM, pH 8.0) at 30 °C for 30 min

^b^The *k*_cat_/*K*_M_ values of CHDA was determined with 10 μM purified *Ac*CO (WT) or its variants and 1–100 mM HMCA in 1 mL air-saturated potassium phosphate buffer (100 mM, pH 8.0) at 30 °C for 30 min

To address this limitation, we considered that changing the interactions between HMCA and the enzyme in the active center may be an efficient strategy to adjust the catalytically active conformation and reduce the accumulation of HMCA. Therefore, the site-screening range was expanded to 8 Å of 4-Cα-OH of HMCA based on variant W2. This change was expected to alter the interactions between 4-Cα-OH of HMCA with the surrounding residues to adjust the catalytically active conformation and consequently reduce the D_(4-Cα-H)-N(5)FAD_ of HMCA. Toward this end, nine residues (W331, T463, N462, W61, F357, N378, Q329, V355, and M359) next to 4-Cα-OH of HMCA were selected and engineered by an iterative saturation mutation approach based on variant W2 (Additional file 1: Fig. S8C, D), resulting in variant W4 (S101A/H351V/N378S/Q329N). The specific activity of variant W4 toward CHDA was 4.21 ± 0.38 U·g^−1^, which represented a 1.5-fold, 1.7-fold, 3.4-fold, 4.4-fold, and 14.5-fold increase compared with the corresponding activities of variants W3, W2, W1*, W1, and WT, respectively (Table 1). As a result, the yield of CHDA increased to 40.1% and the yield of HMCA decreased to 56.6% (the titer decreased to 2.83 mM) (Fig. 4).

The kinetic parameters of the variants were determined, which are listed in Tables 1 and 2. The *K*_M_^CHDA^ (5.24 ± 0.28 mM) and *K*_M_^HMCA^ (1.12 ± 0.19 mM) values of variant W4 were 5.1-fold and 13.7-fold lower than those of WT, respectively, which indicated that the affinity with HMCA and CHDM of W4 was significantly enhanced. The *k*_cat_^CHDA^ (0.94 ± 0.043 min^−1^) and *k*_cat_^HMCA^ (2.20 ± 0.43 min^−1^) values of variant W4 increased by 21.8-fold and 13.8-fold, respectively, which indicated that variant W4 display a higher activity of primary alcohol oxidation. Accordingly, the *k*_cat_/*K*_M_^CHDA^ (0.18 mM^−1^·min^−1^) and *k*_cat_/*K*_M_^HMCA^ (1.96 mM^−1^·min^−1^) values increased by 112.5-fold and 196-fold, respectively. Although the selectivity of CHDA did not show complete reversal, the yield of CHDA and the R_CHDA/HMCA_ increased to 40.1% and 0.72, respectively (Fig. 4). To evaluate the industrial-scale application potential of variant W4, CHDA was synthesized at the 3 L scale. Under the optimum conditions [72 g·L^−1^ (0.5 M) CHDM, 30 g·L^−1^ whole cell catalyst (wet *E. coli*), 5% (v/v) DMSO, 0.1 g·L^−1^ catalase, 0.1 M air-saturated potassium phosphate buffer, pH 8.0, and 30 °C], the titer of CHDA reached up to 29.6 g·L^−1^ in 12 h, with 42.2% yield and 2.5 g·L^–1^·h^–1^ space–time yield (STY) **(**Additional file 1: Figs. S10 and S11).

**Table 2:** Kinetic constants of *Ac*CO and its variants in production of HMCA

Strains	^a^Specific activity^HMCA^ (U·g^−1^)	*K*_M_^HMCA^ (mM)	*k*_cat_^HMCA^ (min^−1^)	^b^*k*_cat_/*K*_M_^HMCA^ (mM^−1^·min^−1^)
WT	1.20 ± 0.13	15.30 ± 0.14	0.16 ± 0.028	0.010
W1(S101A)	2.34 ± 0.22	13.45 ± 0.23	0.37 ± 0.064	0.027
W1*(H351V)	2.43 ± 0.22	13.36 ± 0.12	0.41 ± 0.027	0.031
W2(S101A/H351V)	5.31 ± 0.27	10.21 ± 0.46	1.31 ± 0.011	0.13
W3(S101A/H351V/N378S)	6.27 ± 0.15	7.63 ± 0.72	1.87 ± 0.12	0.24
W4(S101A/H351V/N378S/Q329N)	8.76 ± 0.46	1.12 ± 0.19	2.20 ± 0.43	1.96

^a^The specific activity of HMCA was determined with 10 μM purified *Ac*CO (WT) or its variants and 5 mM CHDM in 1 mL air-saturated potassium phosphate buffer (100 mM, pH 8.0) at 30 °C for 30 min

^b^The *k*_cat_/*K*_M_ values of HMCA was determined with 10 μM purified *Ac*CO (WT) or its variants and 1–100 mM CHDM in 1 mL air-saturated potassium phosphate buffer (100 mM, pH 8.0) at 30 °C for 30 min

### Structural analysis and performance enhancement mechanism of variant W4

**Fig. 5:**
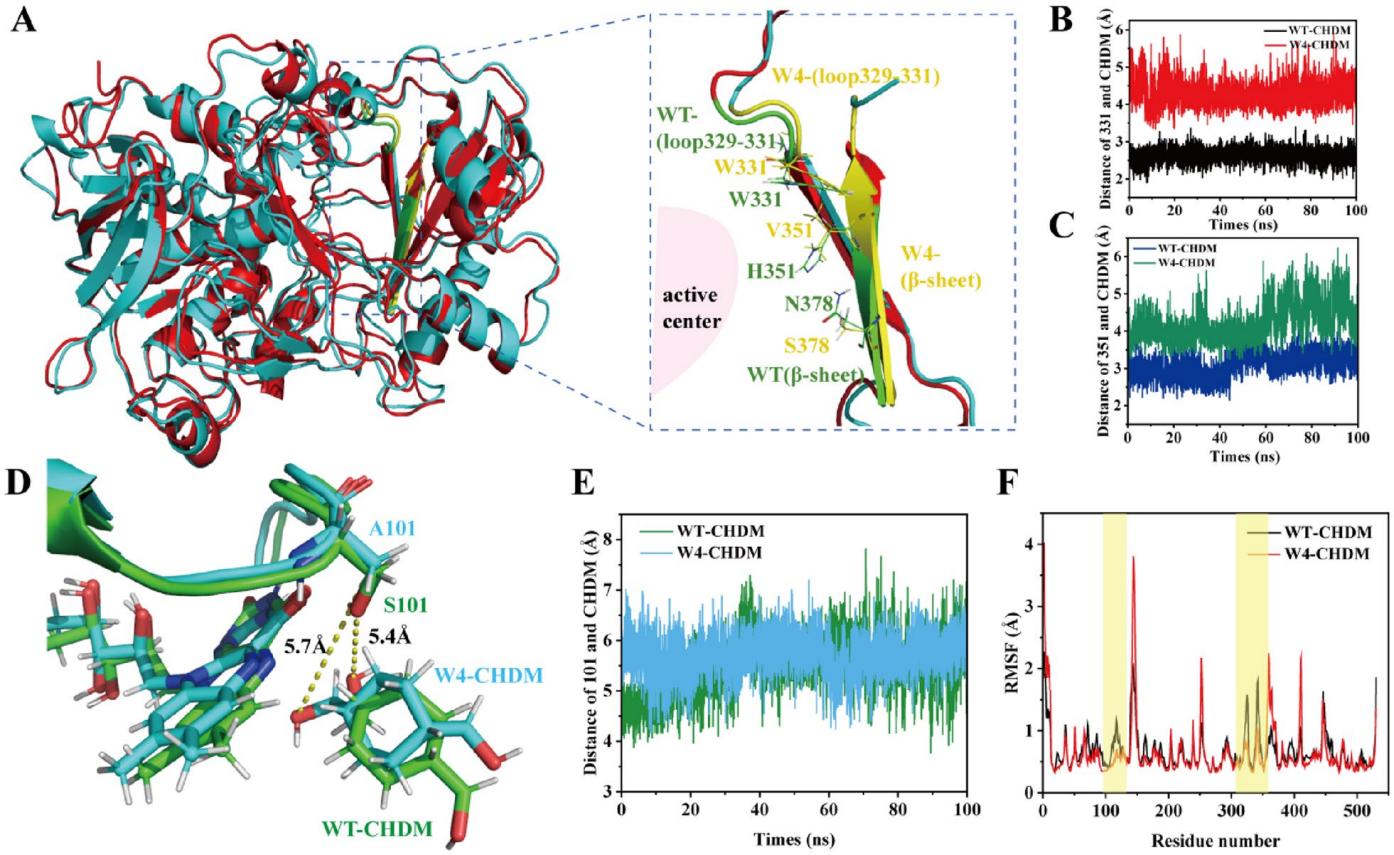
Structure alignments of WT and variant W4. **A** Structure alignments of WT-CHDM and variant W4-CHDM in loop 329–332 and β-sheet. WT-CHDM is shown in green, variant W4-CHDM is shown in yellow. **B** Panel shows the distance of W331 and CHDM in both the WT and variant W4. **C** Panel shows the distance of residue 351 and CHDM in both the WT and variant W4. **D** Structure alignments of WT-CHDM and variant W4-CHDM in loop 100–103, WT-CHDM is shown in green, variant W4-CHDM is shown in blue. **E** Panel shows the distance of residue 101 and CHDM in both the WT and variant W4. **F** Panel shows the RMSF values for WT-CHDM and variant W4-CHDM complex

To elucidate the mechanisms of the improved catalytic efficiency of the variant W4, the structure alignments of the WT-CHDM and W4-CHDM complex were elucidated, demonstrating that the structures are very similar with only slight differences in loop 100–103, loop 329–332, and the β-sheet, which is below the 4-Cα-OH of CHDM. The S101A mutation is located in loop 100–103 and is close to the catalytic center. The mutations H351V and N378S lie on the β-sheet, which is below the 4-Cα-OH of CHDM. In contrast, the mutation Q329N is located on loop 329–332, which is far away from the catalytic center (Fig. 5A, D). Therefore, interaction analysis was performed to better understand the loop swing. In the WT-CHDM, steric interactions occur between H351 and CHDM, and this interaction is weakened when mutated to the nonpolar residue V351, due to an increase in the distance between the side chain of V351 and the C atom of CHDM to 4.2 Å, which causes a dent in the steric effect (Fig. 5C). Moreover, the mutations N378S and H351V showed synergism, and further weakened the interactions between 4-Cα-OH of CHDM and the surrounding residues (Fig. 4). The residue S101 interacts with 1-Cα-OH of CHDM, and the W331 side chain forms an edge-to-face interaction with 4-Cα-OH of CHDM; thus, with the mutation of S101 to A101 and the mutation of Q329 to N329, the movement of loop 100–103 and loop 329–332 toward CHDM were restricted, and the distance between A101 and W331 with 1-Cα-OH and 4-Cα-OH of CHDM increased from 5.4 Å to 5.7 Å and from 2.6 Å to 4.3 Å, respectively (Fig. 5B, D and E). The root mean square fluctuation (RMSF) values of the residues were determined for both the WT and variant W4 using trajectory analysis, which revealed that the RMSF values of loop 100–103 and loop 329–332 of W4 were lower than those of WT (Fig. 5F). This result was consistent with the higher stability of loop 100–103 and loop 329–332. Therefore, the weakened flexibility of loop 100–103 and loop 329–332 in the variant W4 may contribute to restricting the inward loop swing, which prevents A101 and W331 from moving closer to 1-Cα-OH and 4-Cα-OH of CHDM and provides an extra space for conformation adjustment. This phenomenon was also observed in the WT-HMCA and W4-HMCA complex (Additional file 1: Fig. S12).

**Fig. 6:**
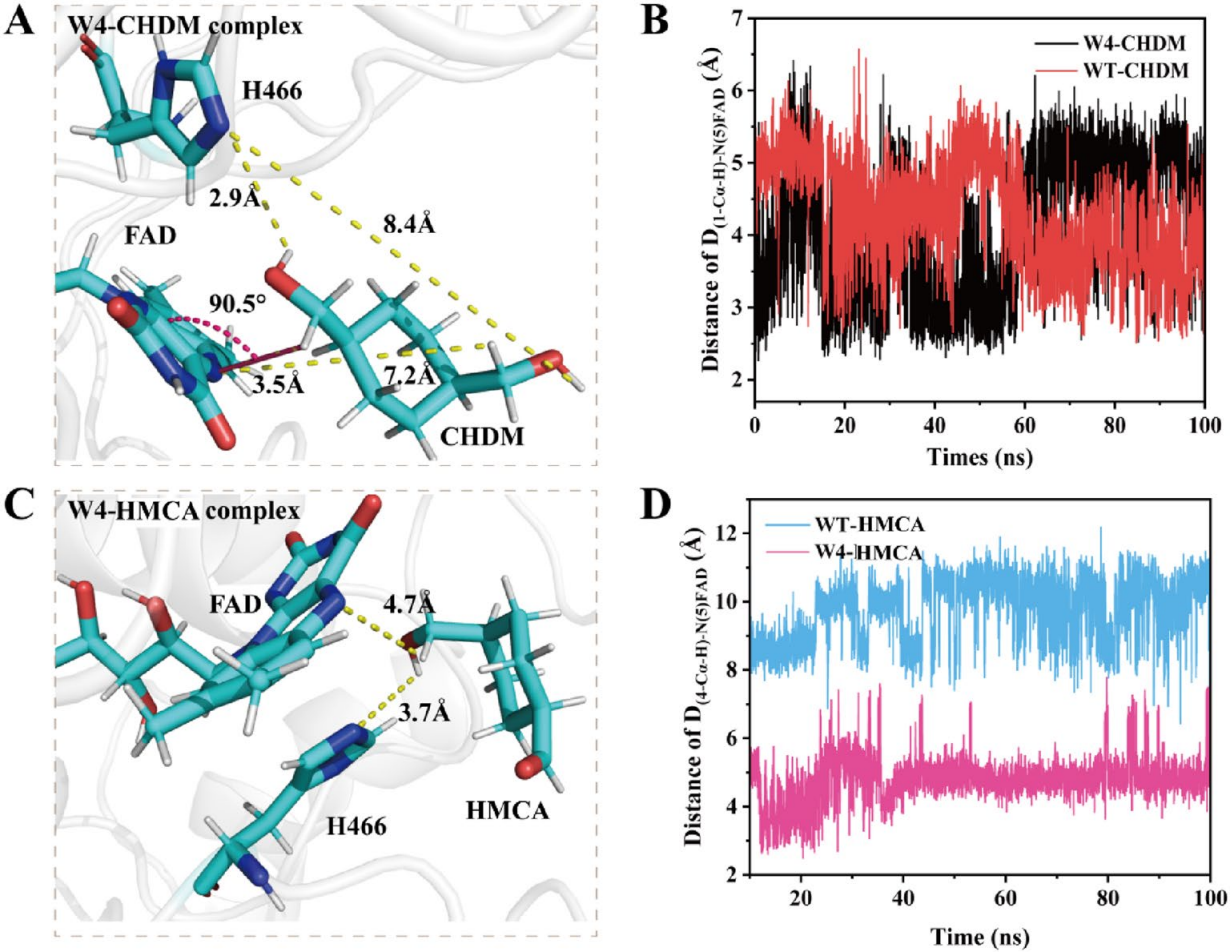
Catalytically active conformation of W4-CHDM and W4-HMCA complex. **A** Catalytically active conformation of W4-CHDM. FAD, H466 and CHDM are shown in cyan. **B** Distance of hydride (H^−^) transfer in WT-CHDM and W4-CHDM complex. **C** Catalytically active conformation of W4-HMCA. FAD, H466 and HMCA are shown in cyan. **D** Distance of hydride (H^−^) transfer in WT-HMCA and W4-HMCA complex

Therefore, the catalytically active conformation of CHDM and HMCA changed substantially in the variant. In the conformation of the W4-CHDM complex, the angle θ_N(10)-N(5)-(1-Cα-OH)_ decreased from 124.1° to 90.5°, thereby approaching 90° (Additional file 1: Fig. S13), in turn leading the D_(1-Cα-H)-N(5)FAD_ and D_(4-Cα-H)-N(5)FAD_ to decrease to 3.5 Å and 7.2 Å, respectively (Fig. 6A, B). The decreased distance in W4-CHDM is beneficial for the hydride (H^−^) transfer and the energy barrier of this step was decreased to 6.2 kcal·mol^−1^ (Fig. 3B). Moreover, the distance of the nucleophilic attack [D_(1-Cα-OH)-N_^ε2^_H466_ and D_(4-Cα-OH)-N_^ε2^_H466_] decreased to 2.9 Å and 8.4 Å, which facilitated the nucleophilic attack from H466 to 1-Cα-OH of CHDM, and consequently increased the catalytic efficiency of CHDM (Additional file 1: Fig. S14). In the conformation of the W4-HMCA complex, 4-Cα-OH of HMCA rotated to become buried in the active center, while 1-CHO of HMCA was anchored to the outside of the binding pocket, and these changes led the D_(4-Cα-H)-N(5)FAD_ to decrease to 4.7 Å, while the D_(4-Cα-OH)-N_^ε2^_H466_ changed to 3.7 Å (Fig. 6C, D). The favorable conformation enhanced more HMCA to convert to CHDA, and the energy barrier of the hydride (H^−^) transfer was decreased to 9.2 kcal·mol^−1^, thereby facilitating the oxidization of 4-Cα-OH for the synthesis of CHDA, while simultaneously reducing HMCA accumulation in the bioconversion broth (Fig. 3B). In summary, the above-mentioned changes in the catalytically active conformation of CHDM and HMCA were beneficial in forming a lower energy barrier, leading to a higher transfer efficiency of hydride (H^−^) and more efficient primary alcohol oxidation by variant W4 to produce CHDA (29.6 g·L^–1^, 42.2% yield, STY 2.5 g·L^–1^·h^–1^).

## Discussion

We here provide the first report of an engineered *Ac*CO variant with improved catalytic efficiency to produce CHDA. Based on the characterized structure, the molecular mechanism of the FAD-dependent primary alcohol oxidation reaction catalyzed by *Ac*CO was elucidated, demonstrating that the unfavourable catalytically active conformation of CHDM and HMCA at the active site, led to an excessively long distance of hydride (H^−^) transfer, which brought about a high energy barrier for hydride (H^−^) transfer and consequently a lower efficiency of primary alcohol oxidation. Thus, a protein engineering strategy was employed to adjust the catalytically active conformation to decrease the hydride (H^−^) transfer distance, which enabled the identification of the optimum variant W4, with 112.5-fold increased catalytic efficiency (*k*_cat_/*K*_M_^CHDA^) compared with that of the WT. Furthermore, the recombinant strain for producing CHDA was successfully established using the best variant (W4), achieving a 29.6 g·L^–1^ CHDA titer with 42.2% yield in a 3 L fermenter.

To elucidate the reason for the low catalytic efficiency in a specific enzyme, three basic aspects should be addressed: structural basis, molecular mechanism, and location within the enzyme (Song et al. 2020). In this study, the catalytically active conformation of CHDM and HMCA and the energy barrier of hydride (H^−^) transfer were elucidated by MD simulations and QM/MM calculations. The power of MD simulations and QM/MM calculations was instrumental in observing the substrate access and energy change, that could not be achieved solely by structural inspection. Moreover, the energy barrier of hydride (H^−^) transfer calculated by QM/MM provided a theoretical basis for the influence of the long distance of hydride (H^−^) transfer on catalytic efficiency in previous studies (Wu et al. 2021; Xu et al. 2019).

For the optimum variant W4, the MD simulation indicated that the weakened interactions between CHDM and HMCA with variant W4 significantly decreased the distance of hydride (H^−^) transfer through adjusting catalytically active conformation in a favorable orientation. As a result, the energy barrier related to the hydride (H^−^) transfer were reduced 7.3 kcal·mol^−1^ and 11.0 kcal·mol^−1^, and thus the catalytic efficiency improved to 0.18 mM^−1^·min^−1^. Compared with other studies based on modification of the residual volume for adjusting the catalytically active conformation of substrates, the changes in interactions can identify mutation hotspots more accurately, guide the construction of small and smart libraries, and ultimately improve the evolution efficiency (Wen et al. 2021). Although *Ac*CO has been identified and engineered by Turner’s group (such as variant S101A), in this study, we elucidated the catalytic mechanism more comprehensively based on QM/MM. The optimal variant W4 (S101A/H351V/N378S/Q329N) was obtained, which achieved the gram-grade preparation of CHDA (Heath et al. 2019).

Finally, using the *Ac*CO variant, an efficient biocatalyst was established for the synthesis of CHDA. Although there are several chemical methods to produce CHDA, the most commonly used methods involve primary alcohol oxidation using a strong oxidizing agent (such as PCC oxidant) (Silbert et al. 2019). However, these methods require complicated post-processing procedures and use of toxic chemical reagents. Furthermore, the use of other biocatalysts such as laccases and ADH is limited owing to their low catalytic efficiency and NAD^+^/cofactor-recycling system (An et al. 2019; Martinez-Montero et al. 2017). Therefore, the biocatalytic process used in this study provides an attractive strategy for the transfer of inexpensive materials into high-value CHDA, and may remarkably facilitate the industrial production of this valuable chemical.

### Supplementary Information

**Additional file 1. **Additional Tables S1–S6, additional Figures S1–S14, and additional materials and methods.

## Data Availability

All data generated or analyzed during this study are included in this article.

## Abbreviations

CHDA: 1,4-Cyclohexanedicarboxaldehyde
CHDM: 1,4-Cyclohexanedimethanol
HMCA: 4-(Hydroxymethyl)cyclohexanecarboxaldehyde
FAD: Flavin adenine dinucleotide
ADH: Alcohol dehydrogenases
AOX: Alcohol oxidases
