# Supplementary material for: Efficient synthesis 1,4-cyclohexanedicarboxaldehyde by an engineered alcohol oxidase
Source: Bioresour Bioprocess. 2022 Aug 13;9(1):80. doi: 10.1186/s40643-022-00570-y (PMC10991250; doi:10.1186/s40643-022-00570-y)
Supplement: Supplementary file 1 — Additional file 1. Additional Tables S1–S6, additional Figures S1–S14, and additional materials and methods. [file 40643_2022_570_MOESM1_ESM.docx]

# Efficient synthesis 1,4-cyclohexanedicarboxaldehyde by an engineered alcohol oxidase

Yaqi Cheng^1^, Wei Song^1^, Xiulai Chen^2^, Cong Gao^2^, Jia Liu^2^, Liang Guo^2^, Meng Zhu^3^, Liming Liu^1,2^, Jing Wu^1,2*^

^1^ School of Life Sciences and Health Engineering, Jiangnan University, Wuxi, Jiangsu 214122, China.

^2^ State Key Laboratory of Food Science and Technology, Jiangnan University, Wuxi, Jiangsu 214122, China

^3^ Wuxi Acryl Technology Co., LTD, Wuxi 214122, China.

* Corresponding author.

Mailing address: School of Life Sciences and Health Engineering, Jiangnan University, 1800 Lihu Road, Wuxi 214122, China.

Fax/Tel: +86-510-85915657

E-mail: wujing@jiangnan.edu.cn (Jing Wu)

**This file includes:**

Table S1 to Table S6

Figure S1 to Figure S14

Additional materials and methods

# Additional Tables

**Table S1.** Strain used for genetic construction

| Strain | origin |
| --- | --- |
| *Ac*CO | *Arthrobacter cholorphenolicus* |
| GOase | *Fusarium graminearum* |
| *Rs*AO | *Rhodococcus triatomae* |
| *Ss*ADH | *Sulfolobus solfataricus* |
| *Brs*ADH | *Brevibacterium sp.* |
| HlADH | Horse liver |
| *Tv*L | *Trametes versicolor* |
| *Bs*L | *Trametes villosa* |
| *Tvi*L | Bacillus subtilis |

**Table S2.** Primers used for genetic construction

| Primer | Sequence (5’-3’) |
| --- | --- |
| *Ac*CO-F | CAGCAAATGGGTCGCGGATCCATGCATATCGATAACATCGAAAATTT |
| *Ac*CO-R | TTGTCGACGGAGCTCGAATTCTTAAGCGAATGATGTGGTCAACTC |
| GOase-F | CGCGGATCCGAATTCGAGCTCATGAAACACCTTTTAACACTCGCTC |
| GOase-R | TGCGGCCGCAAGCTTGTCGACTCACTGAGTAACGCGAATCGTC |
| *Rs*AO-F | ATGGGTCGCGGATCCGAATTCATGTCCGAAAAGCGATTCGA |
| *Rs*AO-R | GCAAGCTTGTCGACTGAGCTCTCAGGCCCCCTTCTTGATCA |
| *Ss*ADH-F | CAGCAAATGGGTCGCGGATCCATGCGTGCAGTTCGCTTGG |
| *Ss*ADH-R | TTGTCGACGGAGCTCGAATTCTTAGGGAATCAAAACTTGGCGA |
| *Brs*ADH-F | CAGCAAATGGGTCGCGGATCCATGAAAGCATCCCTTGCAACC |
| *Brs*ADH-R | TTGTCGACGGAGCTCGAATTCTCAAAATGACGTAATCACACTGCG |
| HlADH-F | ATGGGTCGCGGATCCGAATTCATGAGCACAGCAGGAAAAGTAATAA |
| HlADH-R | GCAAGCTTGTCGACGGAGCTCTCAAAACGTCAGGATGGTACGG |
| *Tv*L-F | ATGGGTCGCGGATCCGAATTCATGGGCAGGTTCTCATCTCTCTG |
| *Tv*L-R | GCAAGCTTGTCGACGGAGCTCTTAGAGGTCGGATGAGTCAAGAGC |
| *Bs*L-F | CAGCAAATGGGTCGCGGATCCATGACACTTGAAAAATTTGTGGATG |
| *Bs*L-R | GCAAGCTTGTCGACGGAGCTCTTATTTATGGGGATCAGTTATATCCATC |
| *Tvi*L-F | CAGCAAATGGGTCGCGGATCCATGGGTCTGCAGCGATTCAG |
| *Tvi*L-R | GCAAGCTTGTCGACGGAGCTCTCACTGGTTAGCCTCGCTCAG |

**Table S3.** Primers used for variants construction

| Primer | Sequence (5’-3’) |
| --- | --- |
| S101-F | TCCAGTCACAATNNKTGTATT |
| S101-R | GAACGCAATACAMNNATTGTG |
| H351-F | GACTTGATGATGNNKTATGGCTCA |
| H351-R | TACTGAGCCATAMNNCATCATCAA |
| Y465-F | CATAATACCGTCNNKCATCCT |
| Y465-R | GCCTACAGGATGMNNGACGGT |
| N510-F | GTGACTGTTNNKCCAAACATC |
| N510-R | AGTGATGTTTGGMNNAACAGT |
| I103-F | CACAATTCCTGTNNKGCGTTC |
| I103-R | CGCCCAGAACGCMNNACAGGA |
| V464-F | ACGCATAATACCNNKTACCATCCT |
| V464-R | TACAGGATGGTAMNNGGTATTATG |
| W331-F | AGCACGCAATGGNNKGAAATC |
| W331-R | GATTTCMNNCCATTGCGTGCT |
| T463-F | AAGACGCATAATNNKGTCTAC |
| T463-R | AGGATGGTAGACMNNATTATG |
| N462-F | CGTAAGACGCATNNKACCGTC |
| N462-R | ATGGTAGACGGTMNNATGCGT |
| W61-F | CAGTTAGATCGCNNKATGGAA |
| W61-R | AAGTAATTCCATMNNGCGATC |
| F357-F | GGCTCAGTACCANNKGATATG |
| F357-R | AGTGTTCATATCMNNTGGTAC |
| N378-F | AGCTTGACCCCTNNKGTTACA |
| N378-R | TGCGTGTGTAACMNNAGGGGT |
| Q329-F | CAAACAAGCACGNNKTGGTGGGAA |
| Q329-R | GATTTCCCACCAMNNCGTGCTTGT |
| V355-F | CATTATGGCTCANNKCCATTT |
| V355-R | CATATCAAATGGMNNTGAGCC |
| M359-F | GTACCATTTGATNNKAACACT |
| M359-R | GCGAAGAGTGTTMNNATCAAA |
| H466A-F | AACACCGTGTACGCGCCGGTG |
| H466A-R | TGTGCCCACCGGCGCGTACAC |

**Table S4.** The specific activities of AOX from different source

| Enzymes | Organisms | Specific activity^CHDA^(U·g^-1^) ^c^ | Specific activity^HMCA^(U·g^-1^) ^c^ |
| --- | --- | --- | --- |
| *Ac*CO^a^ | *Arthrobacter cholorphenolicus* | 0.11±0.026 | 1.2±0.13 |
| GOase^b^ | *Fusarium graminearum* | 0.024±0.014 | 0.64±0.16 |
| *Rs*AO^a^ | *Rhodococcus triatomae* | 0.093±0.031 | 0.98±0.11 |

^a^The specific activity of CHDA and HMCA was determined with 10 μM purifed enzymes and 2 mM HMCA and CHDM in 1 mL air-saturated potassium phosphate buffer (100 mM, pH 8.5) at 30 °C for 25 min.

^b^The specific activity of CHDA and HMCA was determined with 10 μM purifed enzymes and 2 mM HMCA and CHDM in 1-mL NaPi buffer (200 mM, pH 7.4) at 25 °C for 30 min.

^c^The products HMCA and CHDA were extracted by dichloromethane and then tested in GC.

**Table S5.** The specific activity of ADH from different organisms

| Enzymes | Organisms | Specific activity^CHDA^(U·g^-1^) ^d^ | Specific activity^HMCA^(U·g^-1^) ^d^ |
| --- | --- | --- | --- |
| *Ss*ADH^a^ | *Sulfolobus solfataricus* | 0.094±0.015 | 1.14±0.17 |
| *Brs*ADH^b^ | *Brevibacterium sp.* | 0.047±0.011 | 0.84±0.12 |
| HlADH^c^ | Horse liver | 0.073±0.015 | 0.96±0.13 |

^a^The specific activity of CHDA and HMCA was determined with 10 μM purifed enzymes, 1 mM NAD^+^ and 2 mM HMCA and CHDM in 1 mL Glycine-NaOH buffer (100 mM, pH 10.5) at 65 °C for 30 min.

^b^The specific activity of CHDA and HMCA was determined with 10 μM purified enzymes, 1 mM NAD^+^ and 2 mM HMCA and CHDM in 1 mL Glycine-NaOH buffer (100 mM, pH 9.0) at 25 °C for 30 min.

^c^The specific activity of CHDA and HMCA was determined with 10 μM purified enzymes, 1 mM NAD^+^ and 2 mM HMCA and CHDM in 1 mL CHES (N-Cyclohexyl-2-aminoethanesulfonic acid) buffer (100 mM, pH 9.0) at 25 °C for 30 min.

^d^The products HMCA and CHDA were extracted by dichloromethane and then tested in GC.

**Table S6.** The specific activity of laccases from different organisms

| Enzymes | Organisms | Specific activity^CHDA^(U·g^-1^) ^c^ | Specific activity^HMCA^(U·g^-1^) ^c^ |
| --- | --- | --- | --- |
| *Tv*L^a^ | *Trametes versicolor* | 0.064±0.013 | 0.83±0.12 |
| *Bs*L^b^ | Bacillus subtilis | n/a | n/a |
| *Tvi*L^b^ | *Trametes villosa* | 0.033±0.017 | 0.43±0.14 |

Note. n/a, not available.

^a^The specific activity of CHDA and HMCA was determined with 10 μM purifed enzymes, 0.5 mM TEMPO and 2 mM HMCA and CHDM in 1 mL NaOAc buffer (50 mM, pH 4.5) at 20 °C for 30 min.

^b^The specific activity of CHDA and HMCA was determined with 10 μM purifed enzymes, 0.5 mM TEMPO and 2 mM HMCA and CHDM in 1 mL sodium citrate buffer (100 mM, pH 4.5) at 20 °C for 30 min.

^c^The products HMCA and CHDA were extracted by dichloromethane and then tested in GC.

# Additional Figures

**
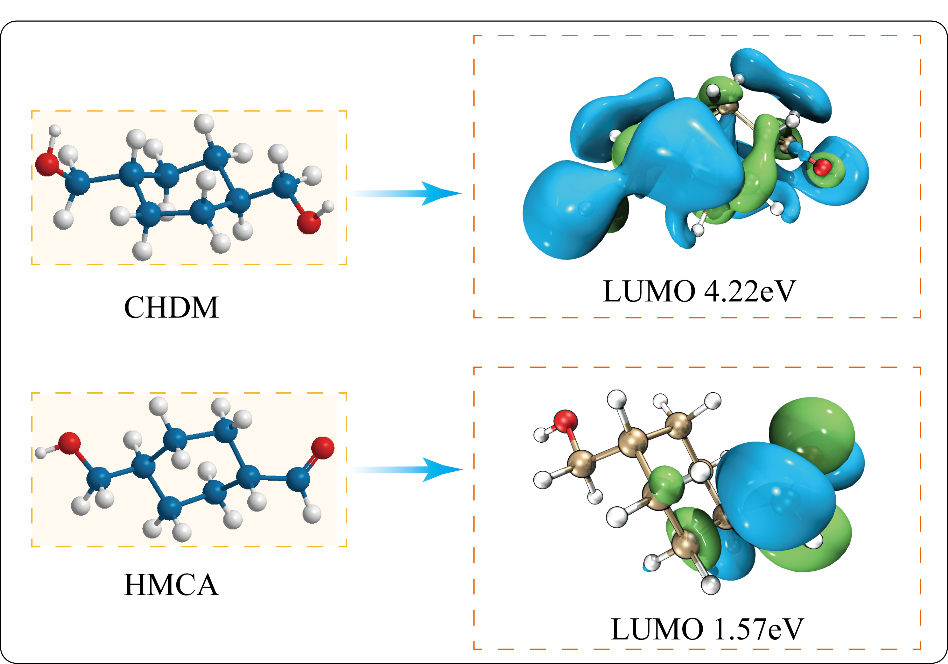
**

**Figure S1.** Orbital calculations of CHDM and HMCA. Blue and green orbitals represent depletion charge and accumulation charge, respectively.


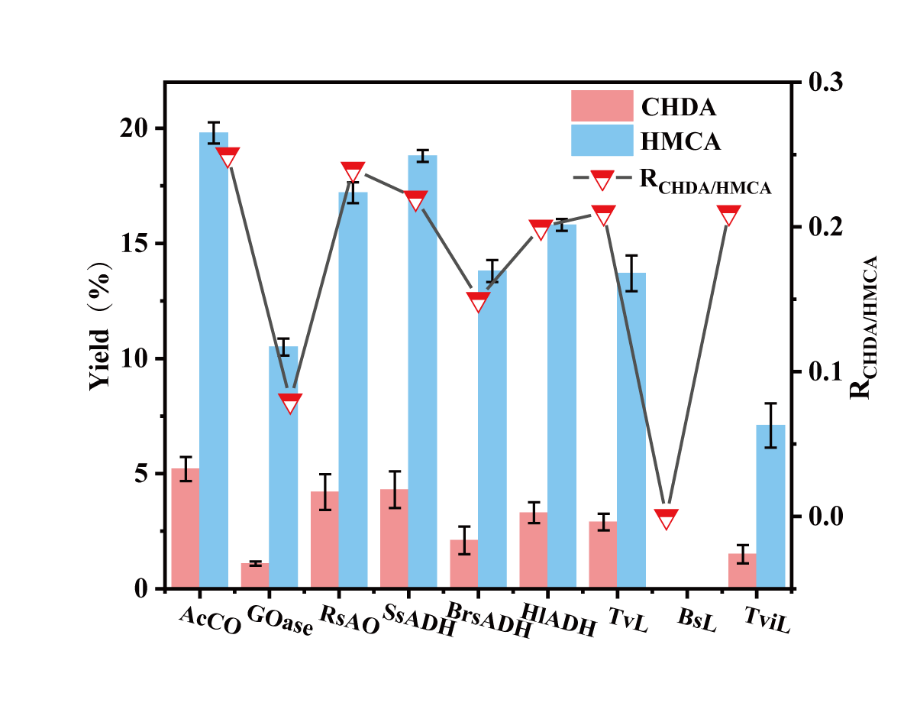


**Figure S2.** The yield of CHDA and HMCA in different source of enzyme.


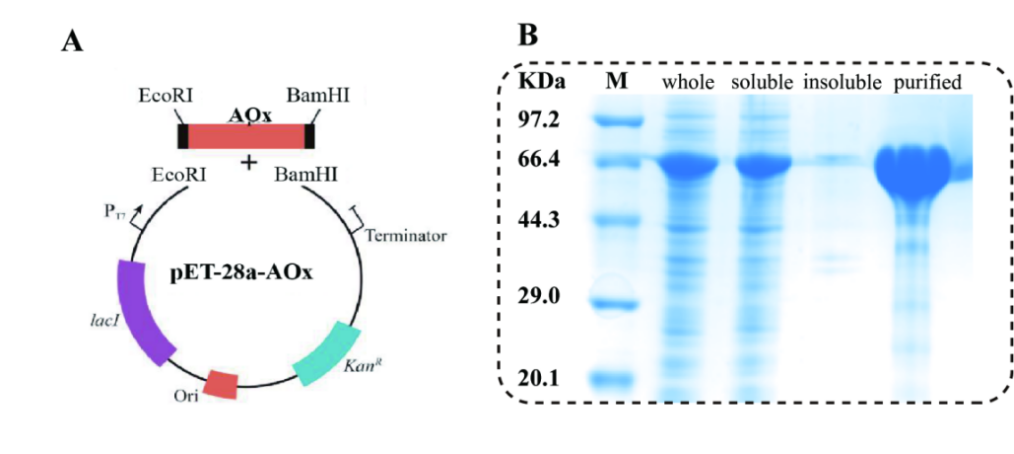


**Figure S3.** Recombinant expression of *Ac*CO. (A) Expression plasmid map; (B) SDS-PAGE analysis of recombinant expression and purification samples.


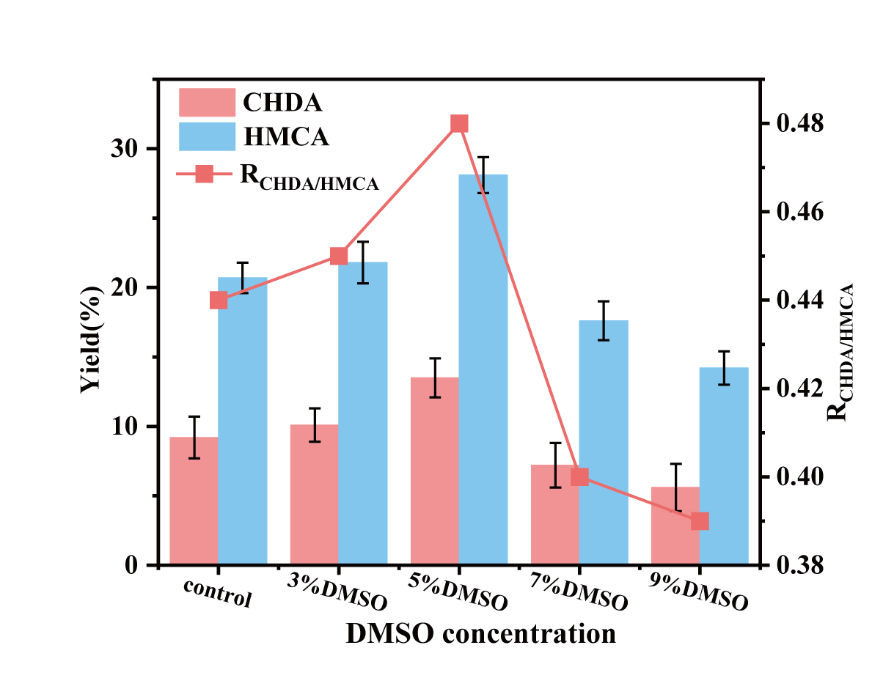


**Figure S4.** Effect of DMSO concentration on the R_CHDA/HMCA_ and the yield of CHDA.


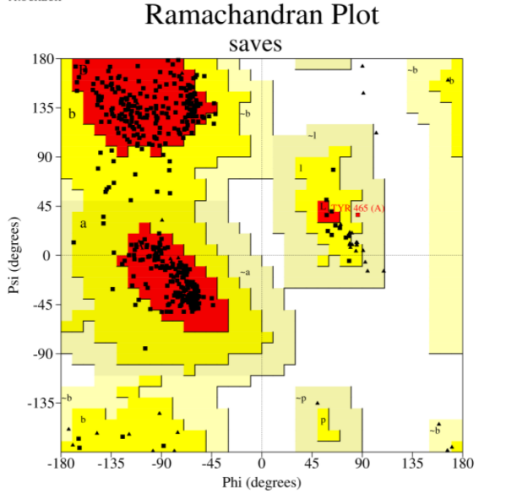


**Figure S5.** Ramachandran plot of *Ac*CO structure.


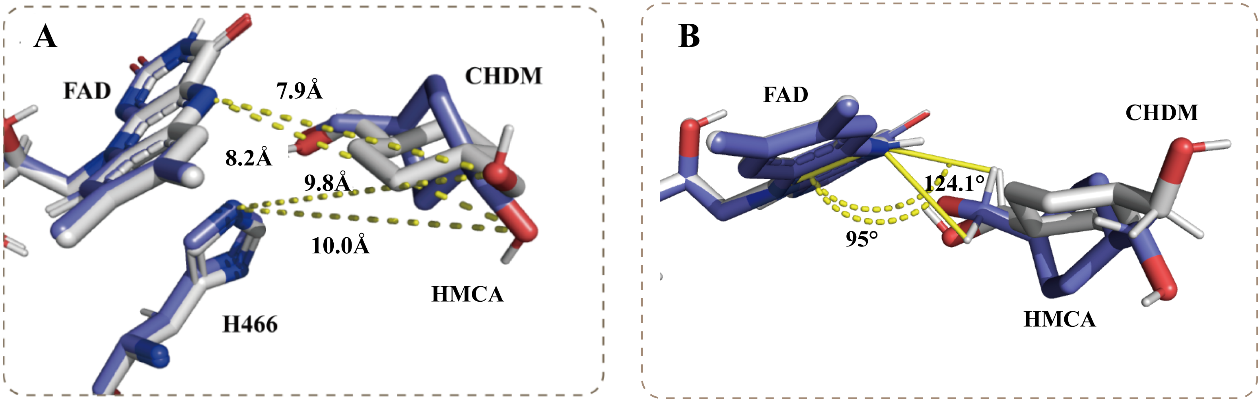


**Figure S6.** Docking model analysis of CHDM and HMCA in WT. (A) A detailed active site view of WT-CHDM (white) and WT-HMCA (purple) complex; (B) The θ_N(10)-N(5)-(1-CHO)_ of HMCA and θ_N(10)-N(5)-(1-Cα-OH)_ of CHDM in WT.


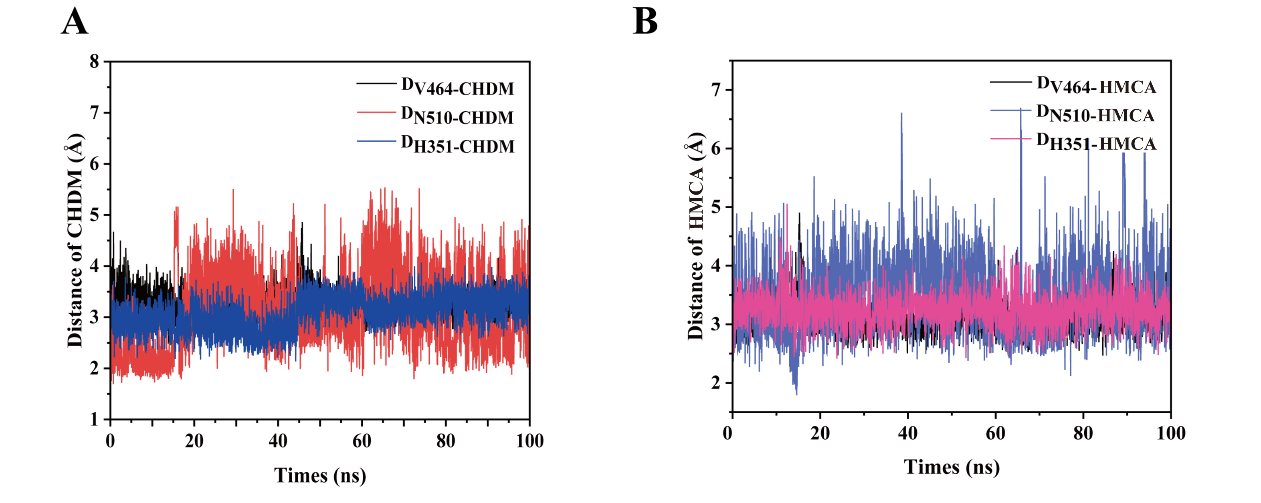


**Figure S7.** MD simulations of WT bound with CHDM and HMCA. (A) MD simulations of WT bound with CHDM. The panel shows the distance of V464, N510, H351 bonding to CHDM; (B) MD simulations of WT bound with HMCA. The panel shows the distance of V464, N510, H351 bonding to HMCA.


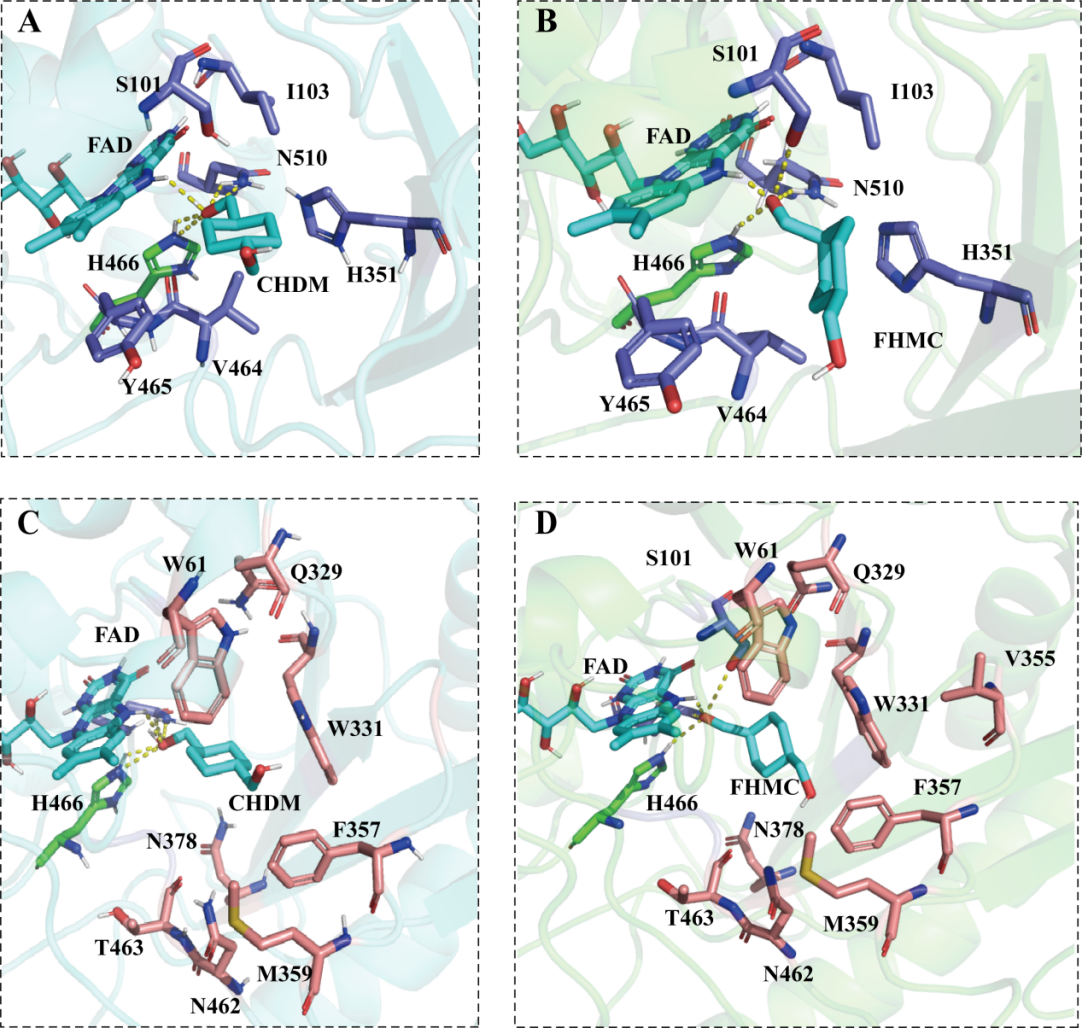


**Figure S8.** The candidate residues of WT-CHDM and WT-HMCA complex. (A) The candidate residues of WT-CHDM complex in active center; (B) The candidate residues of WT-HMCA complex in active center, the candidate residues are shown in purple, FAD and CHDM or HMCA are shown in cyan, H466 is shown in green. (C) The candidate residues of WT-CHDM complex next the 4-Cα-OH of CHDM; (D) The candidate residues of WT-HMCA complex next the 4-Cα-OH of HMCA**,** the candidate residues are shown in pink, FAD and CHDM or HMCA are shown in cyan, H466 is shown in green.


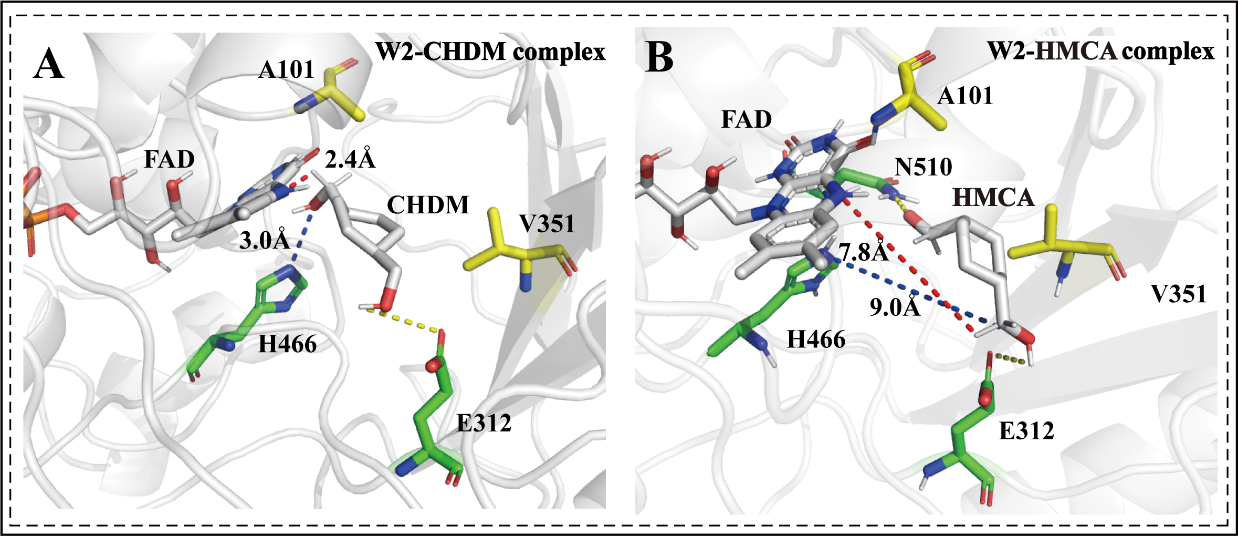


**Figure S9.** The docking models of W2-CHDM and W2-HMCA complex. (A) The docking model of W2-CHDM. The FAD and CHDM are shown in white, residues H466 and E312 are shown in green. The hydrogen bond between CHDM and residues E312 is shown in yellow dash lines. The blue dash lines denote D_(4-Cα-OH)-N_^ε2^_H466_. The red dash lines denote D_(4-Cα-H)-N(5)FAD_. (B) The docking model of W2-HMCA. The red dash lines denote D_(4-Cα-OH)-N_^ε2^_H466_. The blue dash lines denote D_(4-Cα-H)-N(5)FAD_.


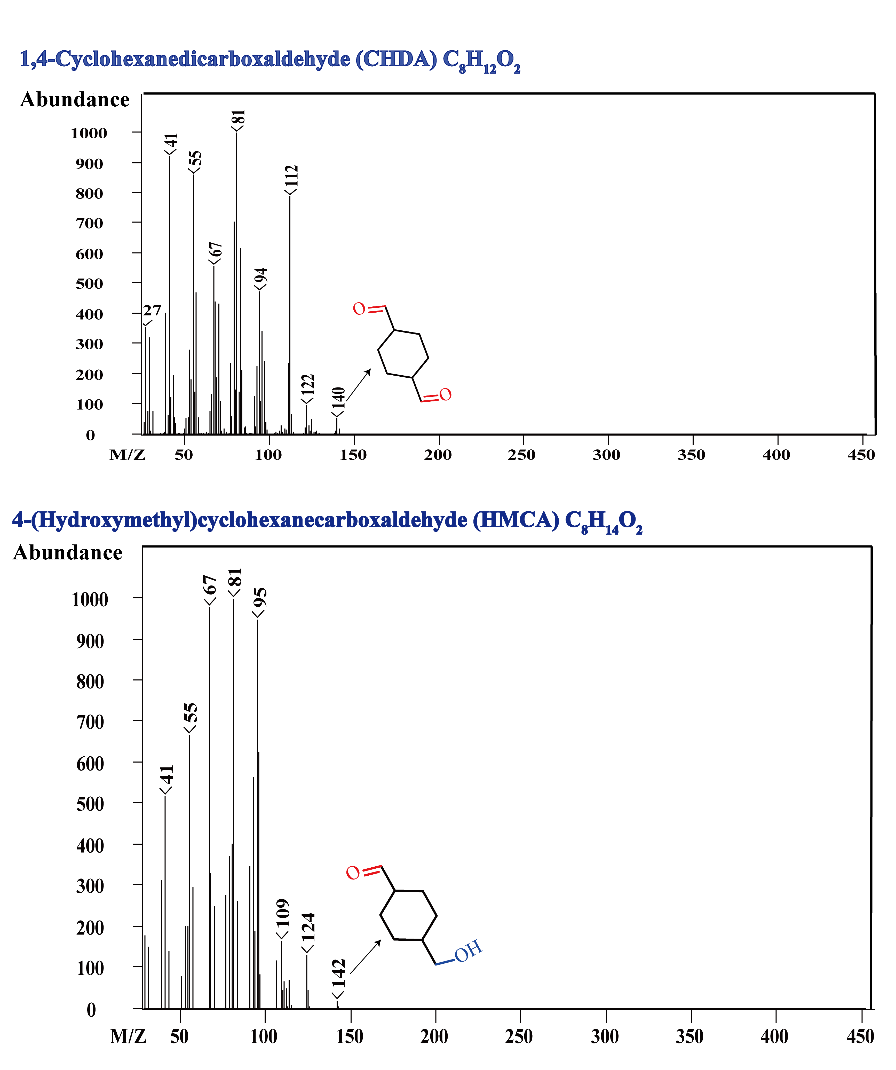


**Figure S10.** The mass spectrum of CHDA and HMCA


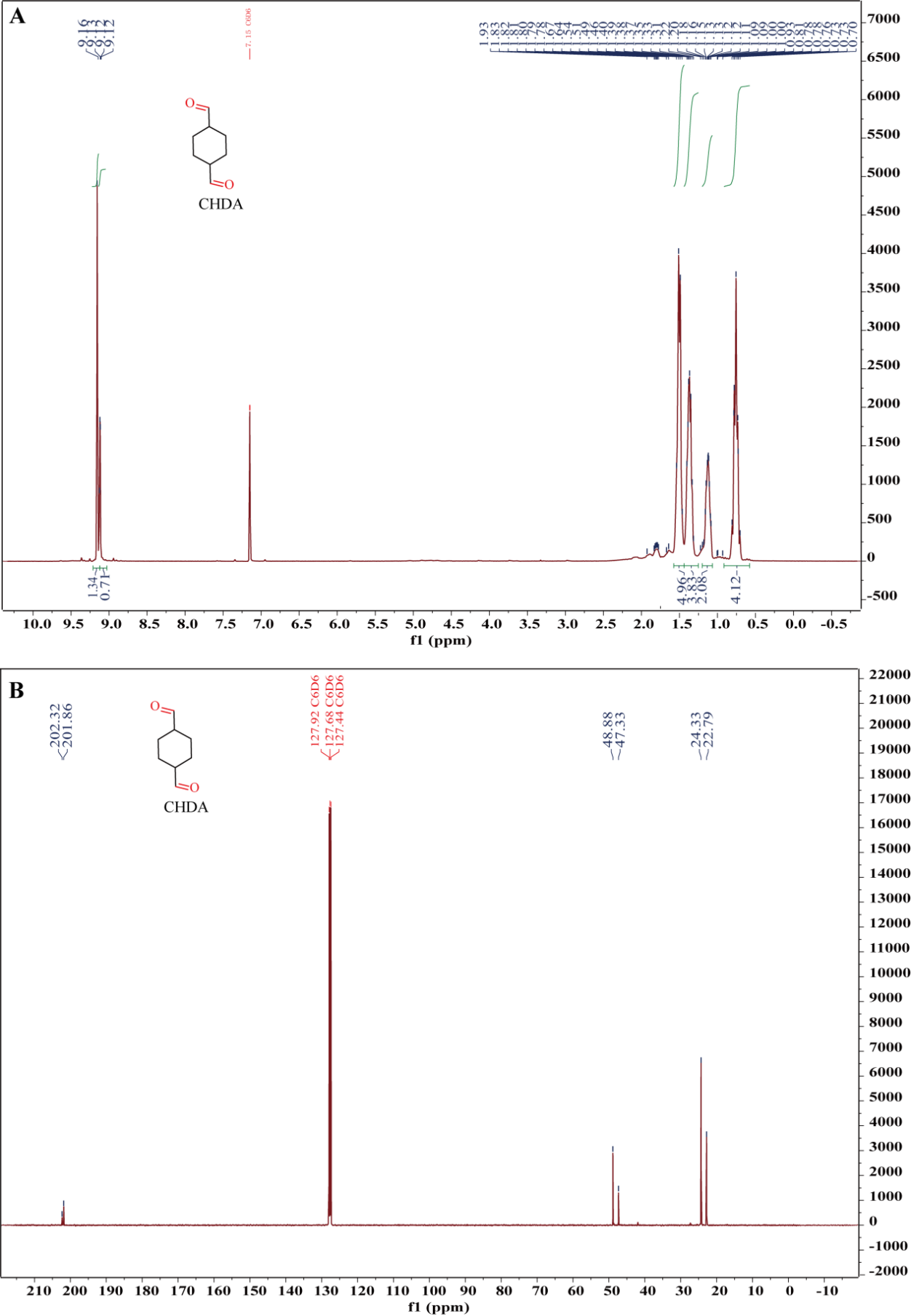


**Figure S11.** NMR spectra of CHDA. (A) ^1^H-NMR spectra of CHDA. (B) ^13^C-NMR spectra of CHDA.


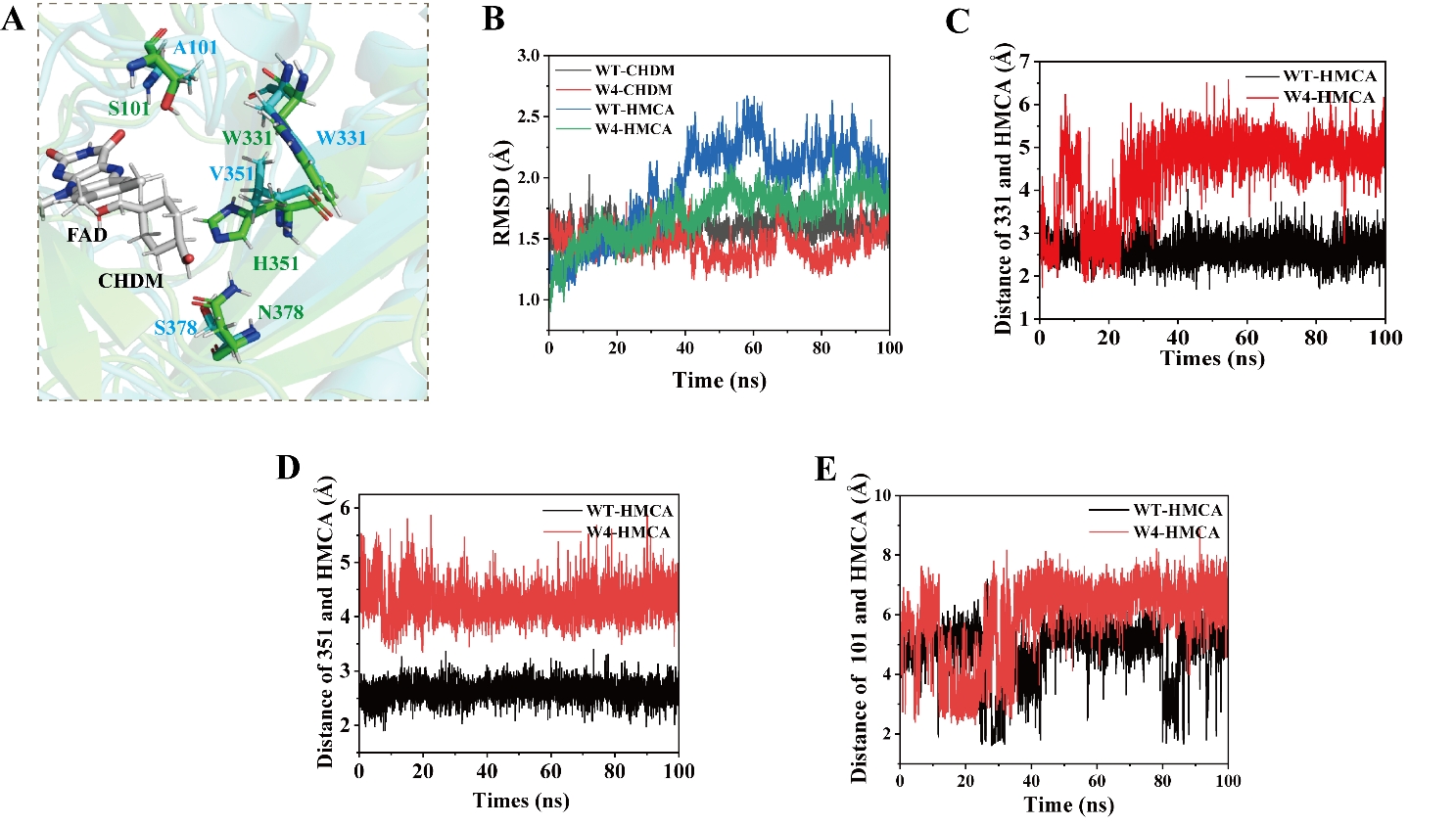


**Figure S12.** Structure alignments of WT-HMCA and W4-HMCA. (A) Structure alignments of WT-HMCA and W4-HMCA, WT-HMCA is shown in green, W4-HMCA is shown in yellow. (B) The panel shows the RMSD values of wild type and variant W4. (C) The panel shows the distance of W331 and HMCA in both WT-HMCA and W4-HMCA complex. (D) The panel shows the distance of residue 351 and HMCA in both WT-HMCA and W4-HMCA complex. (E) The panel shows the distance of residue 101 and HMCA in both WT-HMCA and W4-HMCA complex.

**Figure S13.** The panel shows the angles of θ_N(10)-N(5)-(1-Cα-OH)_ in WT-CHDM and W4-CHDM complex.

**Figure S14.** The panel shows the distance of D_(4-Cα-OH)-N_^ε2^_H466_ and D_(4-Cα-H)-N(5)FAD_ in W4-CHDM complex.

# Additional materials and methods

**The protein sequence of *Ac*CO**

MHIDNIENLSDRGFDYVVIGGGSAGAAVAARLSEDPDVSVALVEAGPDDRNIPEILQLDRWMELLESGYDWDYPIEPQENGNSFMRHARAKVMGGCSSHNSCIAFWAPREDLDEWESKYGATGWNAANAWPLYKRLETNQDAGPDAPHHGDSGPVHLMNVPPADPSGVALLDACEEAGIPRARFNTGTTVVNGANFFQINRRGDGTRSSSSVSYIHPIIERDNFTLLTGLRARQLVFDADKRCTGVEVVDGAFGRTHRLTARHEVILSTGAIDSPKLLMLSGIGPAEHLAQHGIEVLVDSPGVGENLQDHPEGVVQFEAKQPMVQTSTQWWEIGIFTPTEDGLDRPDLMMHYGSVPFDMNTLRHGYPTTENGFSLTPNVTHARSRGTVRLRSRDFRDKPMVDPRYFTDPEGHDMRVMVAGIRKAREIAAQPAMSAWTGRELSPGVGAQTDEELQDYIRKTHNTVYHPVGTVRMGADDDGMSPLDARLRVKGVTGLRVADASVMPEHVTVNPNITVMMIGERCADLIKADYAGADALEEKELTTSFA

**Construction of the variants**

The recombination of variants were constructed by KOD polymerase through a whole-plasmid polymerase chain reaction (PCR) protocol with the main primers listed in **Table S3**. PCR system (25 µL) comprised KOD Plus-Neo (0.5 µL), 10×KOD Plus-Neo Buffer (2.5 µL), dNTP mix (2.5 µL), MgSO_4_ (1.5 µL), template (100-150 ng), corresponding primers (20 µM with 0.5 µL), and sterilized water. The PCR product was then digested by *DpnI* for 3 h at 37°C and then transformed into *Escherichia coli* BL21(DE3) for subsequent DNA sequencing (GENEWIZ, China) or screening.

**Shaking culture**

A single colony of recombinant *E. coli* strain was cultivated for 10-12 h (37℃) in Luria-Bertani (LB) medium (10 g·L^-1^ peptone, 5 g·L^-1^ yeast extract, and 10 g·L^-1^ NaCl; pH 7.0) with appropriate antibiotics (kanamycin) and used as the inoculum (1%). The culture was then transferred into 150 ml Terrific Broth (TB) medium (24 g·L^-1^ yeast extract, 12 g·L^-1^ tryptone, 5 g·L^-1^ glucose, 2.31 g·L^-1^ KH_2_PO_4_, and 16.43 g·L^-1^ K_2_HPO_4_; pH 7.0) containing appropriate antibiotics in a 500 mL flask. When the OD_600_ of the culture broth reached 0.6-0.8, isopropyl β-D-1-thiogalactopyranoside (IPTG) was added to a final concentration of 0.4 mM to induce gene expression. The cells were inducted at 16°C for 16 h and collected by centrifugation (12,000 *× g*, 5 min). Then, the cell pellets were resuspended in an appropriate buffer to the desired density for biotransformation and protein purification (Liu et al. 2021).

**Fermentation culture**

Additional larger fermentations were conducted in a 3 L fermentation system (INFORS HT Labfors, Switzerland) with an air flow rate of 1.5 vvm and a stirrer speed of 600 rpm. The pH was maintained at 7.0 by automatically feeding concentrated carbon and nitrogen resources (400 g·L^-1^ glucose, 100 g·L^-1^ yeast extract, and 25 g·L^-1^ tryptone). Enzyme expression was induced at 16°C with 0.4 mM IPTG at an optical density of 4 at 600 nm. The cell pellets were collected for preparative biotransformation after 16 h induction ([Qian et al. 2020](#_ENREF_3)).

**Screening of site**-**saturation mutagenesis library for enhanced catalytic efficiency**

The single colonies in culture dishes were randomly picked and cultured into 300 µL LB medium with 1% (v/v) kanamycin in 96-deepwell plates and shaken at 37°C for 12 h. Then they were 1:10 diluted into 450 µL TBA medium (12 g·L^-1^ tryptone, 24 g·L^-1^ yeast extract, 5 g·L^-1^ glycerin, 0.5 g·L^-1^ glucose, 4 g·L^-1^ lactose, 3.3 g·L^-1^ (NH_4_)_2_SO_4_, 6.8 g·L^-1^ KH_2_PO_4_, 7.1 g·L^-1^ NaHPO_4_·12H_2_O, 0.15 g·L^-1^ MgSO_4_) in new 96-deepwell plates. After shaking at 37°C for 3 h (for cell growth), the temperature was decreased to 16°C for 16 h. Then the cells were harvested and freeze-thawed. Next, the cells were resuspended with 500 µL 100 mM air-saturated potassium phosphate buffer (pH 8.0) and 5 mM CHDM was added in 96-deep well plates (containing 0.1 g·L^-1^ catalase, 5% v/v DMSO). After reaction at 30°C for 12 h, 300 µL of reaction mixture was extracted by dichloromethane and layered with centrifugation at 12,000 *× g* for 10 min. Take the underlying organic phase and measure the titer by GC (Liu et al. 2021).

**Enzyme assay**

The specific activity of enzyme toward CHDA or HMCA were measured using purified enzymes. (1) The specific activity of *Ss*ADH toward CHDA or HMCA was determined with 10 μM purifed *Ss*ADH, 1 mM NAD^+^ or 2 mM HMCA and CHDM in 1 mL glycine-NaOH buffer (100 mM, pH 10.5) at 65°C for 30 min; (2) the specific activity of *Brs*ADH toward CHDA or HMCA was determined with 10 μM purified *Brs*ADH, 1 mM NAD^+^ and 2 mM HMCA or CHDM in 1 mL glycine-NaOH buffer (100 mM, pH 9.0) at 25°C for 30 min; (3) the specific activity of HlADH toward CHDA or HMCA was determined with 10 μM purified HlADH, 1 mM NAD^+^ and 2 mM HMCA or CHDM in 1 mL CHES (N-Cyclohexyl-2-aminoethanesulfonic acid) buffer (100 mM, pH 9.0) at 25°C for 30 min; (4) The specific activity of *Tv*L toward CHDA or HMCA was determined with 10 μM purifed *Tv*L, 0.5 mM TEMPO and 2 mM HMCA and CHDM in 1 mL sodium acetate (NaOAc) buffer (50 mM, pH 4.5) at 20°C for 30 min; (5) The specific activity of *Bs*L and *Tvi*L toward CHDA or HMCA was determined with 10 μM purifed enzymes, 0.5 mM TEMPO and 2 mM HMCA or CHDM in 1 mL sodium citrate buffer (100 mM, pH 4.5) at 20°C for 30 min. The reaction mixture were extracted by dichloromethane, centrifuged at 12,000 *× g* for 10 min, and analyzed by GC. One unit of *Ac*CO activity (U) was calculatedas the amount of enzyme producing 1 μM of CHDA or HMCA in 1 min (Tan et al. 2021).

The protein concentration was determined by the Bradford protocol, using bovine serum albumin as the standard. All experiments were repeated three times.

**References**

Liu KF, Chen XL, Zhong YL, Gao C, Hu GP, Liu J, Guo L, Song W, Liu LM (2021) Rational design of a highly efficient catalytic system for the production of PAPS from ATP and its application in the synthesis of chondroitin sulfate. Biotechnol Bioeng 118(11): 4503-4515. https://doi.org/10.1002/bit.27919.

Qian Y, Lu C, Liu J, Song W, Chen X, Luo Q, Wu J (2020) Engineering protonation conformation of L-aspartate-alpha-decarboxylase to relieve mechanism-based inactivation. Biotechnol Bioeng*,* 117(6): 1607-1614. <https://doi.org/10.1002/bit.27316>.

Tan X, Zhang S, Song W, Liu J, Gao C, Chen XL, Liu LM, Wu J (2021) A multi-enzyme cascade for efficient production of d-p-hydroxyphenylglycine from l-tyrosine. Bioresour Bioprocess 8(1):41. <https://doi.org/10.1186/s40643-021-00394-2>.
